# Preparation of a 4′‐Thiouridine Building‐Block for Solid‐Phase Oligonucleotide Synthesis

**DOI:** 10.1002/cpz1.878

**Published:** 2023-09-25

**Authors:** Caecilie M. M. Benckendorff, Yogesh S. Sanghvi, Gavin J. Miller

**Affiliations:** ^1^ Centre for Glycoscience Keele University Keele Staffordshire United Kingdom; ^2^ Lennard‐Jones Laboratory, School of Chemical and Physical Sciences Keele University Keele Staffordshire United Kingdom; ^3^ Rasayan Inc. Encinitas California

**Keywords:** 4′‐thionucleoside, protecting group, phosphoramidite, thiosugar

## Abstract

Starting from a commercially available thioether, we report a nine‐step synthesis of a 4′‐thiouridine phosphoramidite building‐block. We install the uracil nucleobase using Pummerer‐type glycosylation of a sulfoxide intermediate followed by a series of protecting group manipulations to deliver the desired phosphite. Notably, we introduce a 3′,5′‐*O*‐di‐*tert*‐butylsilylene protecting group within a 4′‐thiosugar framework, harnessing this to ensure regiospecific installation of the 2′‐*O*‐silyl protecting group. We envisage this methodology will be generally applicable to other 4′‐thionucleosides and duly support the exploration of their inclusion within related nucleic acid syntheses. © 2023 The Authors. Current Protocols published by Wiley Periodicals LLC.

**Basic Protocol 1**: (2R,3S,4R)‐2,3‐*O*‐Isopopropylidene‐5‐*O*‐*tert*‐butyldiphenylsilyl‐1‐(4‐sulfinyl)cyclopentane: Sulfoxidation

**Basic Protocol 2**: 2′,3′‐*O*‐Isopropylidene‐5′‐*O*‐*tert*‐butyldiphenylsilyl‐4′‐thiouridine: Pummerer glycosylation

**Basic Protocol 3**: 4′‐Thiouridine: Deprotection

**Basic Protocol 4**: 2′‐*O*‐*tert*‐Butyldimethylsilyl‐3′,5′‐di‐*tert*‐butylsiloxy‐4′‐thiouridine: 2′,3′,5′‐*O*‐silylation

**Basic Protocol 5**: 2′‐*O*‐*tert*‐Butyldimethylsilyl‐4′‐thiouridine: Selective 3′‐5′‐desilylation

**Basic Protocol 6**: 2′‐*O*‐*tert*‐Butyldimethylsilyl‐5′‐*O*‐dimethoxytrityl‐4′‐thiouridine: 5′‐*O*‐dimethoxytritylation

**Basic Protocol 7**: 2′‐*O*‐*tert*‐butyldimethylsilyl‐3′‐*O*‐[(2‐cyanoethoxy)(*N*,*N*‐diisopropylamino)phosphino]‐5′‐*O*‐dimethoxytrityl‐4′‐thiouridine: 3′‐*O*‐phosphitylation

## INTRODUCTION

**Figure 1:**
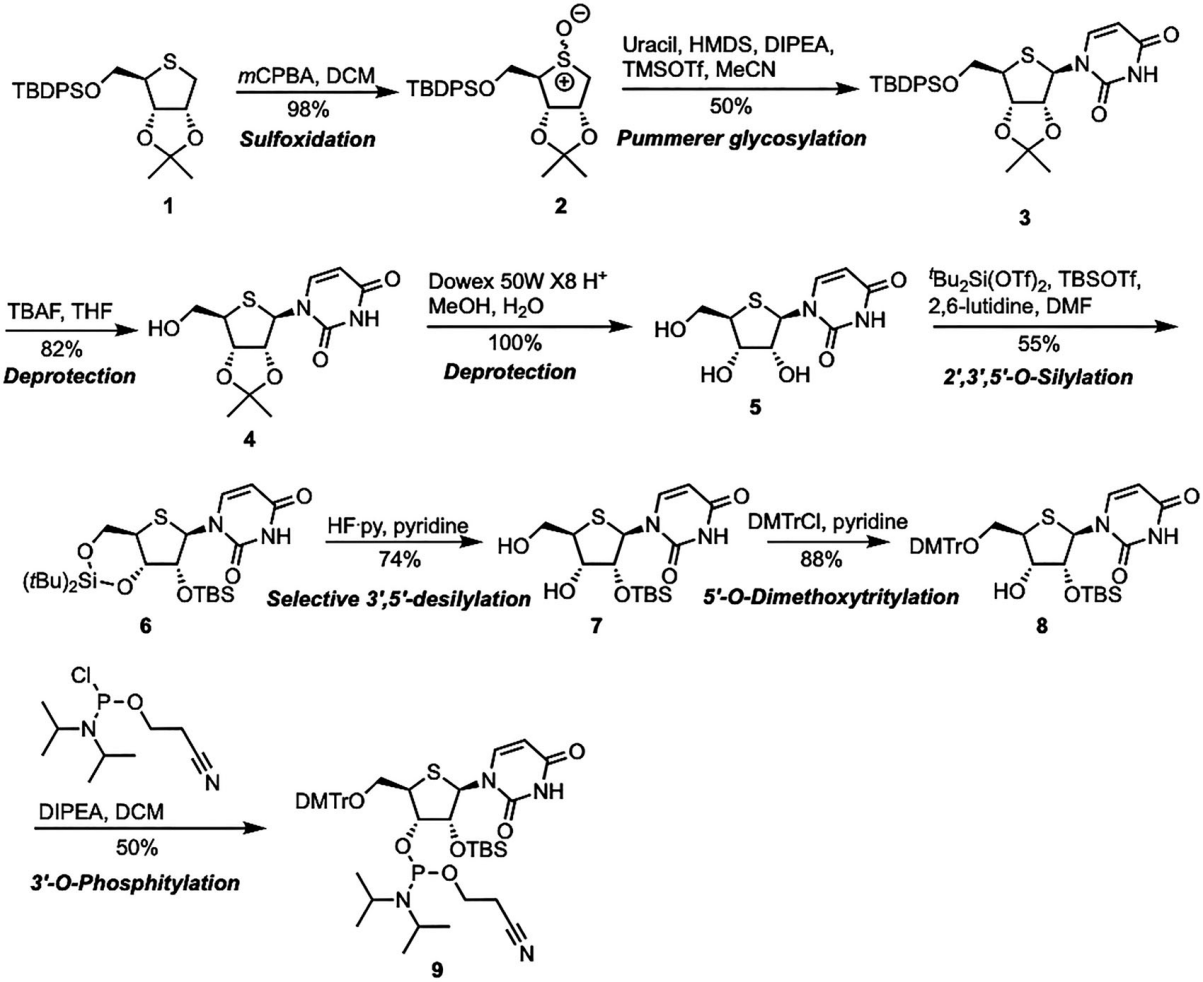
Synthesis of 2‘‐*O*‐*tert*‐butyldimethylsilyl‐3’‐*O*‐[(2‐cyanoethoxy)(*N,N*‐diisopropylamino)phosphino]‐5’‐*O*‐dimethoxytrityl‐4’‐thiouridine. Protecting group abbreviations: TBPDS: *tert*‐butyldiphenylsilyl; ^*t*^Bu2Si(OTf)2: Di‐*tert*‐butylsilyl bis(trifluoromethanesulfonate) TBSOTf: *tert*‐butyldimethylsilyl trifluoromethanesulfonate; di‐*tert*‐butylsilyl bis(trifluoromethanesulfonate) bis(trifluoromethanesulfonate); DMTrCl: 4,4’‐dimethoxytrityl chloride.

Exploration toward chemically synthesizing 4′‐thionucleosides began in the 1960s, with the first reported synthesis of 4′‐thioadenosine in 1964 (Reist et al., 1964). Over the ensuing decades, many examples surrounding the synthesis and evaluation of this nucleoside mimetic have emerged (Dentmon et al., 2020; Dukhan et al., 2005; Dyson et al., 1991; Haraguchi et al., 2019, 2002; Secrist et al., 1991; van Draanen et al., 1996; Yoshimura et al., 1997b, 1997a, 1996), largely within the context of their potential for further development within medicinal chemistry programs (Cosgrove & Miller, 2022; Guinan et al., 2020). Although an excitingly diverse number of 4′‐thionucleoside analogues have now been synthesized and evaluated against a range of viral and oncology targets, a therapeutic has not yet been conferred. Notwithstanding this, the furanosyl replacement of oxygen with larger and less electronegative sulfur has also enabled exploration of this motif within related nucleic acid sequences. Examples here include the following: 1) incorporation of 4‐thioribose within canonical nucleosides, noting conformational changes within related RNA sequences following solid‐phase synthesis (Haeberli, 2005); 2) improved thermal stability and reduced susceptibility toward nucleases for 2′‐modified‐4′‐thioRNA (Takahashi et al., 2009); 3) improved RNA interference in mammalian cells using 4′‐thio‐modified siRNA (Dande et al., 2006); 4) 4′‐thio‐LNA/BNA building blocks introduced within oligonucleotide sequences, seeking to provide new motifs in the advent of mRNA‐based vaccines (Maeda et al., 2021); 5) preparation and exploration of nucleotide triphosphates of 4′‐thionucleosides for SELEX (systematic evolution of ligands by exponential enrichment) (Kato, 2005). Accordingly, access to appropriate 4′‐thionucleoside substrates for the purpose of oligonucleotide synthesis is of continued interest, and in relation to our own programs (Benckendorff et al., 2023, 2022), we have recently developed a reliable and robust synthesis method delivering multi‐gram access to 4′‐thiouridine **5** and derivatives therefrom (Guinan et al., 2022a, 2022b). From this, we wished to gain entry to a 4′‐thiouridine phosphoramidite, appropriately protected for solid‐phase nucleic acid synthesis. Reported herein is our synthesis of this material (Fig. 1).

## Basic Protocol 1 (2R,3S,4R)‐2,3‐*O*‐ISOPOPROPYLIDENE‐5‐*O*‐*TERT*‐BUTYLDIPHENYLSILYL‐1‐(4‐SULFINYL)CYCLOPENTANE 2: SULFOXIDATION

Starting from commercially available thioether **1**, stereoselective attachment of uracil to give 4′‐thiouridine analogue **3** was achieved in 50% yield over two steps from **1**, *via* a Pummerer‐type glycosylation of sulfoxide intermediate **2** (Fig. 1). Next, removal of the 5′‐*O*‐TBDPS group using TBAF afforded 2′,3′‐bis‐*O*‐acetonide protected **4** in 82% yield. Free nucleoside **5** was then obtained in quantitative yield, following 2′,3′‐bis‐*O*‐acetonide hydrolysis using H_2_O with a Dowex 50W X8 H^+^ resin. A subsequent two‐step, one‐pot protection furnished fully silylated **6** in 55% yield was followed by regioselective desilylation of the 3′,5′‐protecting group using HF∙pyridine, yielding 2′‐*O*‐TBS protected nucleoside **7** in 74% yield. Subsequent 5′‐*O*‐tritylation afforded key intermediate **8** in 88% yield (based on 20% recovery of **7**), whereafter phosphitylation at the 3′‐position with 2‐cyanoethyl di‐isopropylchlorophosphoramidite delivered the desired 4′‐thiouridine phosphoramidite building block **9** in 50% yield.

### Materials

Thioether **1**, >95% (offered by Sapala Organics Pvt. Ltd. India)
Dichloromethane (anhydrous, dried over 4‐Å molecular sieves, Sigma‐Aldrich)
*meta‐*Chloroperoxybenzoic acid (*m*‐CPBA), ≤77% (Sigma‐Aldrich)
Hexane, ≥95% (Thermo Fisher Scientific)
Ethyl acetate (EtOAc), ≥99.5% (Sigma‐Aldrich)
Nitrogen gas (passed through a Drierite^®^ drying column)
Sodium bicarbonate (NaHCO_3_), ≥99.7% (Sigma‐Aldrich)
Magnesium sulfate (MgSO_4_) (Thermo Fisher Scientific)
Uracil, ≥99.0% (Sigma‐Aldrich)
Hexamethyldisilazane (HMDS), ≥99% (anhydrous, Sigma‐Aldrich)
Trimethylchlorosilane (TMSCl), 98% (Alfa Aesar)
Acetonitrile (HPLC grade, anhydrous, dried over 3‐Å molecular sieves, Sigma‐Aldrich)
*N*,*N‐*Diisopropylethylamine (DIPEA), 99.5% (Sigma‐Aldrich)
Trimethylsilyl trifluoromethanesulfonate (TMSOTf), 99% (Sigma‐Aldrich)
Silica gel (Sigma‐Aldrich, technical grade, pore size 60 Å, 230 to 400 mesh particle size, 40 to 63 μm particle size)
Tetrahydrofuran (THF, anhydrous, dried over 4‐Å molecular sieves, Thermo Fisher Scientific)
Tetrabutylammonium fluoride (TBAF, 1 M in THF, Sigma‐Aldrich)
DOWEX^®^ 50W X8 H^+^ (Sigma‐Aldrich)
*N*,*N*‐Dimethylformamide (DMF) (Thermo Fisher Scientific, extra dry over molecular sieves, AcroSeal^®^)
2,6‐Lutidine, 98% (Sigma‐Aldrich)
Di‐*tert*‐butylsilyl bis(trifluoromethanesulfonate) (*^t^* Bu_2_Si(OTf)_2_), 97% (Sigma‐Aldrich)
*tert*‐Butyldimethylsilyl trifluoromethanesulfonate (TBDMSOTf), 98% (Sigma‐Aldrich)
4,4′‐Dimethoxytrityl chloride (DMTrCl), recrystallized from hexane (Sigma‐Aldrich)
Pyridine, 99.5% (Thermo Fisher Scientific, extra dry over molecular sieves, AcroSeal^®^)
2‐cyanoethyl di‐isopropylchlorophosphoramidite, 95% (Thermo Fisher Scientific)
Round‐bottom flasks
  25‐ml
  50‐ml
  100‐ml
  500‐ml
500‐ml multinecked round bottom flask
Magnetic stirrer bars
Rubber septa
Stirrer hot plates
Balloons
Vacuum/nitrogen manifold (Connected to vacuum pump, Welch CRVpro 4)
Plastic Luer lock syringes
  1‐ml
  3‐ml
  6‐ml
  12‐ml
  24‐ml
Disposable Luer lock needles
Separatory funnels
  250‐ml
  500‐ml
Conical flasks
  250‐ml
  500‐ml
Rotary evaporator
Silica gel TLC plates (Merck)
Condensers
Chromatography columns
UV lamp
Falcon^™^ tubes
Test tubes
Cannula
Eppendorfs
Ice/NaCl bath

1 Add 2.2 g of **1** (5.1 mmol, 1.0 equiv.) to a 100‐ml round‐bottom flask equipped with a magnetic stirrer bar and stopper the flask with a rubber septum.
2 Degas the reaction vessel (see recipe).
3 Add 51 ml of anhydrous dichloromethane to dissolve starting material.
4 With stirring, cool the flask using an ice/NaCl bath (−10°C to −18°C) for 10 min.
5 Remove the septum from the flask and add *m*‐CPBA (1.1 g, 5.1 mmol, 1.0 equiv.) in one portion, quickly replacing the septum.
  NOTE: The amount of m‐CPBA used was calculated based on 77% purity.
6 Allow the reaction mixture warm to 0°C for 1 hr, with continuous stirring.
7 Monitor the reaction using TLC (see recipe) and elute in 1:1 hexane/EtOAc. The reaction mixture should show complete consumption of the starting material to two spots with lower R_f_. If there is starting material present, leave the reaction mixture stirring for a further hour and monitor the reaction again via TLC.
8 Keeping the flask at 0°C, remove the nitrogen‐filled balloon and septum and quench the reaction mixture by careful addition of saturated aqueous NaHCO_3_ (20 ml; see recipe).
9 Transfer the mixture to a 250‐ml separatory funnel, rinsing the round‐bottom flask with dichloromethane (3 × 15 ml); collect the organic phase in a conical flask.
10 Extract the aqueous phase a further two times with dichloromethane (2 × 50 ml), placing the organic layer in the conical flask containing the first organic layer. Discard the aqueous phase.
11 Place the combined organic layers into the separatory funnel and wash with H_2_O (50 ml). Discard the aqueous layer.
12 Transfer the organic layer to a clean conical flask and dry it over MgSO_4_ for 20 min.
13 Filter the MgSO_4_, rinsing the conical flask with dichloromethane (3 × 20 ml); collect the solvent in a 500‐ml round‐bottom flask and concentrate on a rotary evaporator. The crude product **2** is used in the next reaction without further purification.
14 Characterize the product **2** (2.2 g, 5.0 mmol, 98%) as a 1:1 mixture of diastereoisomers with sulfur and a colorless syrup using TLC, ^1^H NMR, and ^13^C NMR.
  The diastereoisomers were not separated and the following assignments are derived from the constituent mixture. Rf = 0.52 and 0.41 (1:1 hexane/EtOAc).
  Observed for both diastereoisomers: ^1^H NMR (400 MHz, CDCl_3_): δ 7.75 − 7.66 (4H, m, ArH), 7.62 . 7.53 (4H, m, ArH), 7.50 − 7.34 (12H, m, ArH), 3.43 − 3.38 (2H, m, H_1a(a)_, H_1a(b)_), 3.30 − 3.24 (2H, m, H_1b(a)_, H_4(b)_).
  Diastereoisomer a: ^1^H NMR (400 MHz, CDCl_3_): δ 5.23 (1H, td, ^3^ *J*_H2‐H1a/H3_ = 5.9 Hz, ^3^ *J*_H2‐H1b_ = 1.7 Hz, H_2_), 4.99 (1H, dd, ^3^ *J*_H3‐H2_ = 5.9 Hz, ^3^ *J*_H3‐H4_ = 1.7 Hz, H_3_), 4.11 (1H, ov. dd, ^2^ *J*_H5a‐H5b_ = 11.2 Hz, ^3^ *J*_H5a‐H4_ = 2.8 Hz, H_5a_), 3.85 (1H, dd, ^2^ *J*_H5b‐H5a_ = 11.2 Hz, ^3^ *J*_H5b‐H4_ = 3.5 Hz, H_5b_), 3.61 (1H, m, H_4_), 1.59 (3H, s, CH_3_), 1.35 (3H, s, CH_3_), 1.04 (9H, s, [C(C*H*)_3_]); ^13^C NMR (101 MHz, CDCl_3_): δ 135.7 (ArC), 135.6 (ArC), 132.0 (ArC), 131.9 (ArC), 130.44 (ArC), 130.37 (ArC), 128.20 (ArC), 128.17 (ArC), 112.6 [*C*(CH_3_)_3_], 86.7 (C_3_), 85.0 (C_2_), 75.0 (C_4_), 62.0 (C_5_), 58.6 (C_1_), 27.0 [C(*C*H_3_)_3_], 26.9 [C(*C*H_3_)_2_], 24.6 [C(*C*H_3_)_2_], 19.26 [*C*(CH_3_)_3_].
  Diastereoisomer b: ^1^H NMR (400 MHz, CDCl_3_): δ 5.17 (1H, td, ^3^ *J*_H2‐H1a/H3_ = 6.4 Hz, ^3^ *J*_H2‐H1b_ = 4.0 Hz, H_2_), 4.83 (1H, dd, ^3^ *J*_H3‐H2_ = 6.4 Hz, ^3^ *J*_H3‐H4_ = 5.2 Hz, H_3_), 4.18 (1H, dd, ^2^ *J*_H5a‐H5b_ = 11.2 Hz, ^3^ *J*_H5a‐H4_ = 4.3 Hz, H_5a_), 3.20 (1H, dd, ^2^ *J*_H1b‐H1a_ = 14.0 Hz, ^3^ *J*_H1b‐H2_ = 4.0 Hz, H_1b_), 1.44 (3H, s, CH_3_), 1.28 (3H, s, CH_3_), 1.08 (9H, s, [*C*(CH_3_)_3_]); ^13^C NMR (101 MHz, CDCl_3_): δ 135.83 (ArC), 135.82 (ArC), 132.9 (ArC), 132.7 (ArC), 130.10 (ArC), 130.05 (ArC), 127.99 (ArC), 127.98 (ArC), 112.9 [*C*(CH_3_)_2_], 82.1 (C_3_), 80.1 (C_2_), 67.6 (C_4_), 58.0 (C_5_), 56.1 (C_1_), 27.3 [C(*C*H_3_)_2_], 26.9 [C(*C*H_3_)_3_], 24.7 [C(*C*H_3_)_2_], 19.32 [*C*(CH_3_)_3_].

## Basic Protocol 2 2′,3′‐*O*‐ISOPROPYLIDENE‐5′‐*O*‐*TERT*‐BUTYLDIPHENYLSILYL‐4′‐THIOURIDINE 3: PUMMERER GLYCOSYLATION

15 Add uracil (1.1 g, 10.2 mmol, 2.0 equiv.) to a 500‐ml three‐necked round‐bottom flask equipped with a magnetic stirrer bar.
16 Place silicon‐greased glass stoppers in two of the three necks and place a silicon‐greased vacuum adapter connected to a vacuum manifold in the remaining neck.
17 Place the round‐bottom flask on a heating mantle and heat the flask to 110°C with stirring.
18 Evacuate and refill the flask with nitrogen three times and evacuate it so that it is under vacuum. Stir the uracil at 110°C under vacuum for 1 hr.
19 Turn the heating mantle off, fill the flask with nitrogen, and let the flask reach room temperature.
20 Under a positive flow of nitrogen, quickly replace the glass stoppers with rubber septa.
21 Flush a condenser with nitrogen to displace any air; replace the vacuum adapter with the condenser and place the vacuum adapter in the top of the condenser.
22 Add anhydrous acetonitrile (17 ml) to the reaction flask through one of the two septa, suspending the uracil.
23 Add anhydrous HMDS (6.4 ml), followed by TMSCl (410 µl, 3.2 mmol, 0.63 equiv.) to the reaction flask through one of the two septa.
24 Heat the reaction mixture to reflux (82°C) and stir until the suspension becomes clear (approx. 1.5 hr).
25 Turn the heating mantle off and let the reaction mixture cool to room temperature before placing in an ice bath (ice/H_2_O); stir the mixture at 0°C for 15 min.
26 Dissolve 2.2 g of **2** (5.0 mmol, 1.0 equiv.) in anhydrous dichloromethane (86 ml) and transfer the solution to the three‐necked reaction flask containing the silylated uracil via cannula. Stir the reaction mixture at 0°C for a further 15 min.
27 Add DIPEA (5.3 ml, 30.6 mmol, 6.0 equiv.) dropwise, followed by TMSOTf (5.6 ml, 30.6 mmol, 6.0 equiv.) dropwise. Stir the reaction mixture at 0°C for 30 min.
28 Monitor the reaction using TLC (see recipe); elute with 1:1 hexane/EtOAc. The reaction mixture should show complete consumption of the starting material to two spots with a higher R_f_.
  NOTE: The lower spot of the product has a similar R_f_ to that of the higher spot of the starting material.
29 Remove the nitrogen inlet and transfer the reaction mixture to a 500‐ml round bottom‐flask and rinse the reaction flask with methanol (3 × 15 ml) into the new flask and concentrate the reaction mixture on a rotary evaporator.
30 Partition the residue between dichloromethane (100 ml) and H_2_O (200 ml) and place in a 500‐ml separatory funnel.
31 Transfer the organic phase to a 500‐ml conical flask.
32 Extract the aqueous phase with dichloromethane (3 × 80 ml) and place the organic layers in the 500‐ml conical flask already containing the organic layer. Discard the aqueous layer.
33 Add MgSO_4_ to the combined organic layers and wait for 20 min.
34 Filter off the organic solvent into a 500‐ml round‐bottom flask and rinse the conical flask with dichloromethane (3 × 20 ml) into a new 500‐ml flask and remove the solvent on a rotary evaporator.
35 Purify the crude material (1:9 mixture of α/β‐anomers, observed via ^1^H NMR) via column chromatography on silica gel (10%‐50% EtOAc/hexane). The desired β‐anomer will elute after the α‐anomer.
36 Transfer the appropriate fractions to a 500‐ml round‐bottom flask, rinsing the test tubes with dichloromethane (3 × 2 ml each); remove the solvent on a rotary evaporator.
37 Transfer the product to a clean 100‐ml round‐bottom flask, rinsing the 500‐ml flask with dichloromethane (3 × 5 ml). Concentrate on a rotary evaporator.
38 Attach an adapter to the high‐vacuum manifold and place the 100‐ml flask containing the product under vacuum for 2 hr.
39 Characterize the product **3** (1.3 g, 2.5 mmol, 50%), a colorless foam, using TLC, ^1^H NMR, ^13^C NMR, and MS.
  R_f_ = 0.50 (1:1 hexane/EtOAc); ^1^H NMR (400 MHz, CDCl_3_): δ 7.70–7.62 (5H, m, ArH, H_6_), 7.49–7.43 (2H, m, ArH), 7.42–7.38 (4H, m, ArH), 6.06 (1H, d, ^3^ *J*_H1’‐H2’_ = 3.1 Hz, H_1’_), 5.47 (1H, d, ^3^ *J*_H5‐H6_ = 8.1 Hz, H_5_), 4.74 (1H, dd, ^3^ *J*_H3’‐H2’_ = 5.6 Hz, ^3^ *J*_H3’‐H4’_ = 2.8 Hz, H_3’_), 4.66 (1H, dd, ^3^ *J*_H2’‐H3’_ = 5.6 Hz, ^3^ *J*_H2’‐H1’_ = 3.1 Hz, H_2’_), 3.89 (2H, app. d, ^3^ *J*_H5’a/H5’b‐H4’_ = 5.7 Hz, H_5’a_, H_5’b_), 3.76 (1H, app. td, ^3^ *J*_H4’‐H5’a/H5’b_ = 5.6 Hz, ^3^ *J*_H4’‐H3’_ = 2.9 Hz, H_4’_), 1.58 (3H, s, CH_3_), 1.31 (3H, s, CH_3_), 1.10 (9H, s, [C(C*H*_3_)_3_]); ^13^C NMR (101 MHz, CDCl_3_): δ 162.3 (C_4_, C=O), 150.0 (C_2_, C=O), 141.1 (C_6_), 135.7 (ArC), 135.5 (ArC), 132.9 (ArC), 132.6 (ArC), 130.1 (ArC), 127.9 (ArC), 112.6 [*C*(CH_3_)_2_], 102.9 (C_5_), 88.9 (C_2’_), 84.1 (C_3’_), 68.2 (C_1’_), 65.1 (C_5’_), 55.6 (C_4’_), 27.5 [C(*C*H_3_)_2_], 27.0 [C(*C*H_3_)_3_], 25.3 [C(*C*H_3_)_2_], 19.4 [*C*(CH_3_)_3_]; HRMS (see Supporting Information): calculated for C_28_H_34_N_2_O_5_SSi [M+H]^+^ 539.2036, found 539.2043.

## Basic Protocol 3 4′‐THIOURIDINE 5: DEPROTECTION

40 Add 1.4 g of **3** (2.7 mmol, 1.0 equiv.) to a clean 25‐ml round‐bottom flask equipped with a magnetic stirrer bar and stopper the flask with a rubber septum.
41 Degas the reaction vessel (see recipe).
42 Add anhydrous THF (6 ml) to the flask, dissolving the starting material.
43 Add TBAF (1.0 M in THF, 2.7 ml, 2.7 mmol, 1.0 equiv.) to the flask and stir the reaction at room temperature for 45 min.
44 Monitor the reaction using TLC (see recipe); elute in 1:1 hexane/EtOAc. The reaction mixture will show complete consumption of the starting material to a lower R_f_.
45 Concentrate the reaction mixture on a rotary evaporator and purify the residue by column chromatography on silica gel, eluting with 0% to 5% methanol/dichloromethane.
46 Transfer the appropriate fractions to a 500‐ml round‐bottom flask, rinsing the test tubes with dichloromethane (3 × 5 ml), and remove the solvent on a rotary evaporator.
47 Transfer the product to a clean 25‐ml round‐bottom flask, rinsing the 500‐ml flask with methanol (3 × 5 ml). Concentrate on a rotary evaporator.
48 Attach an adapter to the high‐vacuum manifold and place the 25‐ml flask containing the product under vacuum for 2 hr.
49 Characterize the product **4** (660 mg, 2.2 mmol, 82%), a colorless foam, using TLC, ^1^H NMR, ^13^C NMR, and MS.
  R_f_ = 0.24 (EtOAc); ^1^H NMR (400 MHz, CDCl_3_): δ 7.77 (1H, dd, ^3^ *J*_H6‐H5_ = 8.2 Hz, H_6_), 5.93 (1H, s, H_1’_), 5.76 (1H, d, ^3^ *J*_H5‐H6_ = 8.1 Hz, H_5_), 4.93–4.89 (2H, m, H_2’_, H_3’_), 3.96 (1H, app. dt, ^2^ *J*_H5’a‐H5’b_ = 11.4 Hz, ^3^ *J*_H5’a‐H4’_ = 4.5 Hz, H_5’a_), 3.90 (1H, app. dt, ^2^ *J*_H5’b‐H5’a_ = 11.0 Hz, ^3^ *J*_H5’b‐H4’_ = 3.9 Hz, H_5’b_), 3.79 (1H, td, ^3^ *J*_H4’‐H5’a/H5’b_ = 4.6 Hz, ^3^ *J*_H4’‐H3’_ = 1.6 Hz, H_4’_), 2.77 (1H, br s, OH), 1.60 (3H, s, CH_3_), 1.33 (3H, s, CH_3_); ^13^C NMR (101 MHz, CDCl_3_): δ 162.7 (C_4_, C=O), 150.3 (C_2_, C=O), 142.4 (C_6_), 112.6 [*C*(CH_3_)_2_], 103.1 (C_5_), 89.3 (C_2’_), 85.6 (C_3’_), 70.8 (C_1’_), 64.2 (C_5’_), 56.0 (C_4’_), 27.7 [C(*C*H_3_)_2_], 25.5 [C(*C*H_3_)_2_]; HMRS (see Supporting Information): calculated for C_12_H_16_N_2_O_5_ [M+H]^+^ 301.0858, found 301.0862.
50 To the round‐bottom flask containing **4** (570 mg, 1.9 mmol, 1.0 equiv.) add a magnetic stirrer bar, followed by methanol (2 ml) and H_2_O (2 ml) to dissolve the starting material.
51 Add DOWEX 50W X8 H^+^ (500 mg) and place a condenser in the neck of the round‐bottom flask.
  NOTE: The resin should be washed with H_2_O, methanol, and dichloromethane prior to use.
52 Heat the reaction mixture to reflux for 4 hr.
53 Monitor the reaction using TLC (see recipe); elute in 9:1 dichloromethane/methanol. The reaction mixture should show complete consumption of the starting material to a lower R_f_. If starting material remains, continue to stir the reaction at reflux until the starting material is consumed. Continue to monitor the reaction using TLC.
54 Allow the reaction mixture to cool to room temperature.
55 Filter off the resin and collect the solvent in a clean 100‐ml round‐bottom flask, rinsing the 25‐ml flask with H_2_O (3 × 5 ml).
56 Remove the solvent on a rotary evaporator, co‐evaporating with toluene (3 × 3 ml).
57 Attach an adapter to the high‐vacuum manifold and place the 100‐ml flask containing the product under vacuum for 2 hr.
58 Characterize the product **5** (495 mg, 1.9 mmol, 100%), a white solid, using TLC, ^1^H NMR, ^13^C NMR, and MS.
  R_f_ = 0.11 (9:1 dichloromethane/methanol); ^1^H NMR (400 MHz, D_2_O): δ 8.18 (1H, d, ^3^ *J*_H6‐H5_ = 8.1 Hz, H_6_), 5.95 (1H, d, ^3^ *J*_H1’‐H2’_ = 5.7 Hz, H_1’_), 5.90 (1H, d, ^3^ *J*_H5‐H6_ = 8.1 Hz, H_5_), 4.32 (1H, dd, ^3^ *J*_H2’‐H1’_ = 5.7 Hz,, ^3^ *J*_H2’‐H3’_ = 3.7 Hz, H_2’_), 4.19 (1H, app. t, ^3^ *J*_H3’‐H2’/H4’_ = 4.1 Hz, H_3’_), 3.87 (1H, dd, ^2^ *J*_H5’a‐H5’b_ = 12.0 Hz, ^3^ *J*_H5’a‐H4’_ = 5.3 Hz, H_5’a_), 3.82 (1H, dd, ^2^ *J*_H5’b‐H5’a_ = 12.0 Hz, ^3^ *J*H_5’b‐H4’_ = 5.5 Hz, H_5’b_), 3.45 – 3.39 (1H, m, H_4’_); ^13^C NMR (101 MHz, D_2_O): δ 166.1 (C_4_, C=O), 152.3 (C_2_, C=O), 143.0 (C_6_), 102.4 (C_5_), 77.4 (C_2’_), 73.4 (C_3’_), 64.3 (C_1’_), 62.4 (C_5’_), 52.2 (C_4’_); HRMS (see Supporting Information): calculated for C_9_H_12_N_2_O_5_S [M+H]^+^ 261.0540, found 261.0530.

## Basic Protocol 4 2′‐*O*‐*TERT*‐BUTYLDIMETHYLSILYL‐3′,5′‐DI‐*TERT*‐BUTYLSILOXY‐4′‐THIOURIDINE 6: 2′,3′,5′‐*O*‐SILYLATION

59 To an oven‐dried 25‐ml round bottom flask equipped with a magnetic stirrer bar, add **5** (395 mg, 1.52 mmol, 1.0 equiv.) and stopper the flask with a rubber septum.
60 Degas the reaction vessel (see recipe).
61 Add 3 ml of anhydrous DMF to the flask, dissolving the starting material (**5**).
62 Add 2,6‐lutidine (1.1 ml, 9.12 mmol, 6.0 equiv.).
63 With stirring, cool the flask using an ice/NaCl bath (−10°C to −18°C) for 10 min.
64 Add *^t^* Bu_2_Si(OTf)_2_ (590 μl, 1.82 mmol, 1.2 equiv.) dropwise to the mixture.
65 Let the reaction mixture warm to 0°C for 1 hr, and continue to stir the reaction at this temperature for a further 5 hr.
66 Monitor the reaction using TLC (see recipe); elute with 9:1 dichloromethane/methanol. The reaction mixture will show complete consumption of the starting material from a lower R_f_ to a higher R_f_. If the starting material (**5**) has not been fully consumed, continue to stir the reaction at 0°C until it is consumed, as observed with TLC monitoring.
  NOTE: Save this aliquot for further TLC analysis.
67 Maintaining the reaction at 0°C, add TBSOTf (700 μl, 3.0 mmol, 2.0 equiv.) dropwise with continued stirring.
68 Allow the reaction mixture to warm to room temperature and stir it overnight (∼16 to 18 hr).
69 Monitor the reaction using TLC (see recipe); elute with 3:2 hexane/EtOAc. The reaction mixture will show complete consumption of the 3′,5′‐*O*‐*^t^* Bu_2_Si‐protected material from a lower R_f_ to a higher R_f_.
  NOTE: Spot the reaction mixture against the aliquot obtained in step 66.
70 Remove the nitrogen‐filled balloon and rubber septum from the reaction flask and quench the reaction by the adding methanol (5 ml).
71 Remove the magnetic stirrer bar and rinse into the reaction flask with methanol.
72 Concentrate the reaction mixture on a rotary evaporator and purify the residue by column chromatography on silica gel, eluting with 10% to 40% EtOAc/hexane.
73 Transfer the appropriate fractions to a clean 250‐ml round‐bottom flask, rinsing the test tubes with dichloromethane (3 × 5 ml); remove the solvent on a rotary evaporator.
74 Transfer the product to a clean 25‐ml round‐bottom flask, rinsing the 250‐ml round‐bottom flask with dichloromethane (3 × 5 ml). Concentrate on a rotary evaporator.
75 Attach an adapter to a high‐vacuum manifold and place the 25‐ml flask containing the product under vacuum for 2 hr.
76 Characterize the product **6** (440 mg, 0.85 mmol, 55%), a white foam, using TLC, ^1^H NMR, ^13^C NMR, and MS.
  R_f_ = 0.56 (3:2 hexane/EtOAc); ^1^H NMR (400 MHz, CDCl_3_): δ 8.19 (1H, br. s, NH), 7.88 (1H, d, ^3^ *J*_H6‐H5_ = 8.2 Hz, H_6_), 5.76 (1H, d, ^3^ *J* = 8.2 Hz, H_5_), 5.72 (1H, d, ^3^ *J*_H1’‐H2’_ = 1.0 Hz, H_1’_) 4.45 (1H, dd, ^2^ *J*_H5’a‐H5’b_ = 10.1 Hz, ^3^ *J*_H5’a‐H4’_ = 4.8 Hz, H_5’a_), 4.23 (1H, app. d, ^3^ *J*_H2’‐H3’_ = 3.2 Hz, H_2’_), 4.11 (1H, dd, ^3^ *J*_H5’b‐H4’_ = 11.4 Hz, ^2^ *J*_H5’b‐H5’a_ = 10.1 Hz, H_5’b_), 3.93 (1H, dd, ^3^ *J*_H3’‐H4’_ = 9.9 Hz, ^3^ *J*_H3’‐H2’_ = 3.2 Hz, H_3’_), 3.77 (1H, ddd, ^3^ *J*_H4’‐H5’b_ = 11.4 Hz, ^3^ *J*_H4’‐H3’_ = 9.9 Hz, ^3^ *J*_H4’‐H5’a_ = 4.8 Hz, H_4’_), 1.04 (9H, s, [C(C*H*_3_)_3_]), 1.02 (9H, s, [C(C*H*_3_)_3_]), 0.95 (9H, s, [C(C*H*_3_)_3_]), 0.24 (3H, s, CH_3_), 0.15 (3H, s, CH_3_); ^13^C NMR (101 MHz, CDCl_3_): δ 162.4 (C_4_, C=O) 150.3 (C_2_, C=O), 140.8 (C_6_), 102.3 (C_5_), 79.9 (C_3’_), 79.1 (C_2’_), 69.1 (C_5’_), 67.3 (C_1’_), 44.6 (C_4’_), 27.6 [C(*C*H_3_)_3_], 27.0 [C(*C*H_3_)_3_], 26.0 [C(*C*H_3_)_3_], 23.1 [*C*(CH_3_)_3_], 20.3 [*C*(CH_3_)_3_], 18.2 [*C*(CH_3_)_3_], −4.0 (CH_3_), −4.9 (CH_3_): HRMS (see Supporting Information): calculated for C_25_H_43_N_2_O_5_SSi_2_ [M+H]^+^ 515.2426, found 515.2419.

## Basic Protocol 5 2′‐*O*‐*TERT*‐BUTYLDIMETHYLSILYL‐4′‐THIOURIDINE 7: SELECTIVE 3′‐5′‐DESILYLATION

77 Transfer **6** (440 mg, 0.85 mmol, 1.0 equiv.) to a clean 50‐ml Falcon tube equipped with a magnetic stirrer and seal the tube with a rubber septum.
  NOTE: HF will etch glass equipment, which is why the reaction was conducted in the plastic Falcon tube.
78 Degas the reaction vessel (see recipe).
79 Add 5.7 ml pyridine to the Falcon tube, dissolving the staring material (**6**).
80 With stirring, cool the reaction mixture using an ice/NaCl bath (−10°C) for 10 min.
81 Add 130 ml of HF/pyridine (70% HF/30% pyridine) dropwise to the mixture.
82 Stir the reaction mixture at −10°C for 3 hr.
83 Monitor the reaction using TLC (see recipe); quench the aliquot with saturated NaHCO_3_. Add a small amount of EtOAc to the Eppendorf and shake well before spotting the organic phase on a TLC plate against the starting material (**6**) and elute with 95:5 dichloromethane/methanol. The reaction mixture should show complete consumption of the starting material from a higher R_f_ to a lower R_f_.
84 Maintaining the reaction mixture at −10°C, remove the nitrogen‐filled balloon and septum from the Falcon tube and quench the reaction by carefully adding saturated NaHCO_3_ (15 ml) with continuous stirring and allow the mixture to warm to room temperature.
  NOTE: Prior to transferring the mixture to a separatory funnel, check that the pH of the aqueous phase is >7.
85 Remove the stirrer bar from the Falcon tube and rinse with EtOAc.
86 Transfer the contents of the Falcon tube to a 100‐ml separatory funnel, rinsing the Falcon tube with EtOAc (2 × 5 ml).
87 Extract the aqueous phase three times with EtOAc (3 × 20 ml); collect the organic layers in a conical flask and discard the aqueous phase.
88 Place the combined organic phases into the separatory funnel and wash once more with saturated NaHCO_3_ (20 ml). Discard the aqueous layer.
89 Wash the organic phase with H_2_O two times (2 × 20 ml). Discard the aqueous layer.
90 Wash the organic phase with brine (20 ml; see recipe). Discard the aqueous layer.
91 Transfer the organic layer to a clean conical flask and dry the organic layer over MgSO_4_ for 20 min.
92 Filter off the MgSO_4_, rinsing the conical flask with EtOAc (3 × 10 ml); collect the solvent in a 250‐ml round‐bottom flask and concentrate on a rotary evaporator.
93 Purify the residue via column chromatography on silica gel, eluting with 0% to 5% methanol/dichloromethane.
94 Transfer the appropriate fractions to a 250‐ml round‐bottom flask, rinsing the test tubes with dichloromethane (3 × 5 ml); remove the solvent on a rotary evaporator.
95 Transfer the product to a clean 25‐ml round‐bottom flask, rinsing the 250‐ml round‐bottom flask with dichloromethane (3 × 5 ml); remove the solvent on a rotary evaporator.
96 Attach an adapter to the high‐vacuum manifold and place the 25‐ml round bottom flask containing the product under vacuum for 2 hr.
97 Characterize the product **7** (240 mg, 0.63 mmol, 74%), a white solid, using TLC, ^1^H NMR, ^13^C NMR, and MS.
  R_f_ = 0.49 (95:5 dichloromethane/methanol); ^1^H NMR (400 MHz, MeOD): δ 8.30 (1H, d, ^3^ *J*_H6‐H5_ = 8.2 Hz, H_6_), 6.03 (1H, d, ^3^ *J*_H1’‐H2’_ = 5.9 Hz, H_1’_), 5.75 (1H, d, ^3^ *J*_H5‐H6_ = 8.1 Hz, H_5_), 4.37 (1H, dd, ^3^ *J*_H2’‐H1’_ = 5.8 Hz, ^3^ *J*_H2’‐H3’_ = 3.5 Hz, H_2’_), 4.08 (1H, app. t, ^3^ *J*_H3’‐H2’/H4’_ = 3.8 Hz, H_3’_), 3.84 (1H, dd, ^2^ *J*_H5’a‐H5’b_ = 11.7 Hz, ^3^ *J*_H5’a‐H4’_ = 4.5 Hz, H_5’a_), 3.79 (1H, dd, ^2^ *J*_H5’b‐H5’a_ = 11.7 Hz, ^3^ *J*_H5’b‐H4’_ = 4.7 Hz, H_5’b_), 3.41 (1H, app. q, ^3^ *J*_H4’‐H3’/H5’a/H5’b_ = 4.4 Hz, H_4’_), 0.90 (9H, s, [C(C*H*_3_)_3_]), 0.10 (3H, s, CH_3_), 0.08 (3H, s, CH_3_); ^13^C NMR (101 MHz, MeOD): δ 166.0 (C_4_, C=O), 152.7 (C_2_, C=O), 143.7 (C_6_), 102.9 (C_5_), 80.9 (C_2’_), 75.3 (C_3’_), 65.6 (C_1’_), 63.7 (C_5’_), 54.0 (C_4’_), 26.2 [C(*C*H_3_)_3_], 18.9 [*C*(CH_3_)_3_], −4.6 (CH_3_), −4.8 (CH_3_); HRMS (see Supporting Information): calculated for C_15_H_25_N_2_O_5_SSi [M−H]^−^ 373.1259, found 373.1259.

## Basic Protocol 6 2′‐*O*‐*TERT*‐BUTYLDIMETHYLSILYL‐5′‐*O*‐DIMETHOXYTRITYL‐4′‐THIOURIDINE 8: 5′‐*O*‐DIMETHOXYTRITYLATION

98 To the 25‐ml round‐bottom flask containing **7** (164 mg, 0.44 mmol, 1.0 equiv.), add DMTrCl (445 mg, 1.3 mmol, 3.0 equiv; see recipe for recrystallization method) and a magnetic stirrer bar and stopper the flask with a rubber septum.
99 Degas the reaction vessel (see recipe).
100 Add 2.3 ml of anhydrous pyridine, dissolving the reagents.
101 Stir the reaction mixture at room temperature for 24 hr.
102 Monitor the reaction using TLC (see recipe); elute with 95:5 dichloromethane/methanol. The reaction mixture will show the formation of a product with a higher R_f_.
  NOTE: The reaction will likely not go to completion; therefore, expect some starting material (**7**) to be present during monitoring.
103 Remove the rubber septum and quench the reaction with methanol (3 ml).
104 Concentrate the mixture on a rotary evaporator and purify the residue via column chromatography on silica gel, eluting with 0% to 5% methanol/dichloromethane.
  NOTE: At this stage, the remaining unreacted **7** can be recovered.
105 Transfer the appropriate fractions to a clean 250‐ml round‐bottom flask, rinsing the test tubes with dichloromethane (3 × 5 ml). Remove the solvent on a rotary evaporator.
106 Transfer the product to a clean 25‐ml round‐bottom flask, rinsing the 250‐ml flask with dichloromethane (3 × 5 ml). Remove the solvent on a rotary evaporator.
107 Attach an adapter to the high‐vacuum manifold and place the 25‐ml round‐bottom flask containing the product under vacuum for 2 hr.
108 Characterize the product **8** (209 mg, 0.31 mmol, 88% based on return of 33 mg, 0.09 mmol of **7**), a white foam, using TLC, ^1^H NMR, ^13^C NMR, and MS:
  R_f_ = 0.74 (95:5 dichloromethane/methanol); ^1^H NMR (400 MHz, CDCl_3_) δ 8.86 (1H, br. s, NH), 8.06 (1H, d, ^3^ *J*_H6‐H5_ = 8.2 Hz, H_6_), 7.46–7.41 (2H, m, ArH), 7.36–7.23 (8H, m, ArH), 6.87–6.83 (4H, m, ArH), 5.98 (1H, d, ^3^ *J*_H1’‐H2’_ = 4.2 Hz, H_1’_), 5.46 (1H, d, ^3^ *J*_H5‐H6_ = 8.2 Hz, H_5_), 4.28 (1H, app. t, ^3^ *J*_H2’‐H1’/H3’_ = 3.9 Hz, H_2’_), 4.16–4.08 (1H, m, H_3’_), 3.80 (6H, ov. s, OCH_3_), 3.56–3.50 (2H, m, H_4’_, H_5’a_), 3.43 (1H, dd, ^2^ *J*_H5’b‐H5’a_ = 9.3 Hz, ^3^ *J*_H5’b‐H4’_ = 3.0 Hz, H_5’b_), 2.34 (1H, d, ^3^ *J*_OH‐H3’_ = 6.2 Hz, OH), 0.91 (9H, s, [C(C*H*_3_)_3_]), 0.16 (3H, s, CH_3_), 0.11 (3H, s, CH_3_); ^13^C NMR (101 MHz, CDCl_3_): δ 162.9 (C_4_, C=O), 158.9 (ArC), 158.8 (ArC), 150.7 (C_2_, C=O), 144.6 (ArC), 141.6 (C_6_), 135.4 (ArC), 130.42 (ArC), 130.35 (ArC), 128.4 (ArC), 128.1 (ArC), 127.3 (ArC) 113.4 (ArC), 102.6 (C_5_), 87.4 [*C*(Ph)(Ph‐OCH_3_)_2_], 80.2 (C_2’_), 75.1 (C_3’_), 65.3 (C_1’_), 63.2 (C_5’_), 55.4 (OCH_3_), 50.6 (C_4’_), 25.8 [C(*C*H_3_)_3_], 18.1 [*C*(CH_3_)_3_], −4.6 (CH_3_), −4.9 (CH_3_); HRMS (see Supporting Information): calculated for C_36_H_45_N_2_O_7_SSi [M+H]^+^ 677.2717, found 677.2710.

## Basic Protocol 7 2′‐*O*‐*TERT*‐BUTYLDIMETHYLSILYL‐3′‐*O*‐[(2‐CYANOETHOXY)(*N,N*‐DIISOPROPYLAMINO)PHOSPHINO]‐5′‐*O*‐DIMETHOXYTRITYL‐4′‐THIOURIDINE 9: 3′‐*O*‐PHOSPHITYLATION

**Figure 2:**
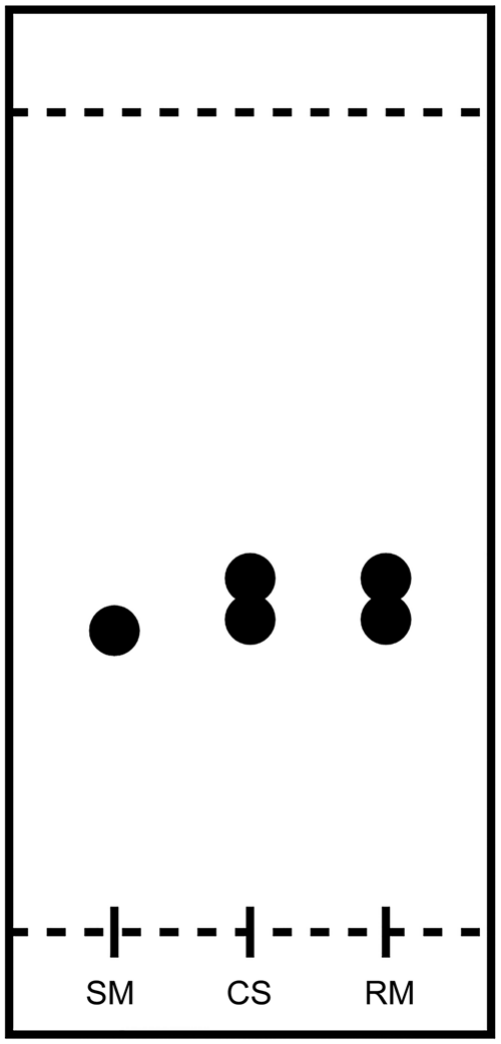
Graphical representation of the TLC plate during reaction monitoring. SM = starting material, CS = co‐spot, and RM = reaction mixture.

109 Add **8** (396 mg, 0.58 mmol, 1.0 equiv.) to a three‐necked 25‐ml round‐bottom flask equipped with a magnetic stirrer bar.
110 Place a silicon‐greased glass stopper in two of the three necks and place a silicon‐greased vacuum adapter connected to a vacuum manifold in the remaining neck.
111 Evacuate and refill the reaction vessel with nitrogen three times.
112 Under a positive pressure of nitrogen, quickly replace the glass stopper with a rubber septum.
113 Add 3.9 ml of degassed anhydrous dichloromethane, dissolving the starting material (**8**).
  NOTE: Degassed solvent was used to minimize oxidation of P(III) materials.
114 Add 410 μl (2.3 mmol, 4.0 equiv.) of degassed anhydrous DIPEA.
115 With stirring, cool the reaction mixture to 0°C using an ice/H_2_O bath for 5 min.
116 Add 2‐cyanoethyl diisopropylchlorophosphoramidite (310 μl, 1.2 mmol, 2.0 equiv.) dropwise to the reaction mixture. Stir the reaction at 0°C for a further 1.5 hr.
117 Monitor the reaction using TLC (see recipe); elute in 1:1 hexane/EtOAc. The reaction mixture should show complete consumption of the starting material to two spots with higher R_f_.
  NOTE: The lower R_f_ of the product (**9**) is very similar to that of the starting material (**8**) and may be difficult to differentiate (see Fig. 2).
  The reaction is in general very quick, and if the spot with a higher R_f_ is present, the reaction has likely gone to completion. The two spots correspond to the two diastereoisomers formed at the phosphorous.

118 Concentrate the reaction mixture on a rotary evaporator and purify the crude material via column chromatography on silica gel neutralized with Et_3_N (0% to 40% EtOAc/hexane + 1% Et_3_N).
119 Transfer the appropriate fractions to a clean 250‐ml round‐bottom flask, rinsing the test tubes with dichloromethane (3 × 5 ml). Remove the solvent on a rotary evaporator.
120 Transfer the product to a clean 25‐ml round‐bottom flask, rinsing the 250‐ml flask with dichloromethane (3 × 5 ml). Remove the solvent on a rotary evaporator.
121 Attach an adapter to the high‐vacuum manifold and place the 25‐ml round bottom flask containing the product under vacuum for 2 hr.
122 Characterize the product **9** (250 mg, 0.29 mmol, 50%), a 1:1 diastereomeric mixture at phosphorous and a white foam, using TLC, ^1^H NMR, and ^13^C NMR.
  The P diastereoisomers were not separated and the following assignments are derived from the constituent mixture: R_f_ = 0.38 and 0.43 (1:1 hexane/EtOAc); ^1^H NMR (400 MHz, CDCl_3_): δ 8.04 (1H, d, ^3^ *J*_H6‐H5_ = 8.2 Hz, H_6_), 7.89 (1H, d, ^3^ *J*_H6‐H5_ = 8.2 Hz, H_6_), 7.52–7.42 (4H, m, ArH), 7.39–7.23 (16H, m, ArH), 6.87–6.81 (8H, m, ArH), 5.94 (1H, d, ^3^ *J*_H1’‐H2’_ = 5.2 Hz, H_1’_), 5.86 (1H, d, ^3^ *J*_H1’‐H2’_ = 4.2 Hz, H_1’_), 5.53 (1H, d, ^3^ *J*_H5‐H6_ = 8.2 Hz, H_5_), 5.50 (1H, d, ^3^ *J*_H5‐H6_ = 8.2 Hz, H_5_), 4.27 (1H, dd, ^3^ *J*_H2’‐P_ = 5.2 Hz, ^3^ *J*_H2’‐H3’_ = 3.2 Hz, H_2’_), 4.24 (1H, dd, ^3^ *J*_H2’‐P_ = 4.3 Hz, ^3^ *J*_H2’‐H3’_ = 3.1 Hz, H_2’_), 4.20–4.11 (2H, m, 2 × H_3’_), 3.93–3.87 (1H, m, CH_2_C*H*_2_CN), 3.80 (12H, ov. s, 4 × OCH_3_), 3.78–3.68 (3H, m, H_4’_, 2 × CH_2_C*H*_2_CN), 3.65–3.45 (8H, m, H_4’_, H_5’a_, H_5’b_, CH_2_C*H*_2_CN, 4 × [C*H*(CH_2_)_3_]), 3.45–3.38 (2H, m, H_5’a_, H_5’b_), 2.62 (2H, t, ^3^ *J*_H‐H_ = 6.1 Hz, C*H*_2_CH_2_CN), 2.44 (2H, td, ^3^ *J*_H‐H_ = 6.3 Hz, ^3^ *J*_H‐P_ = 1.9 Hz, C*H*_2_CH_2_CN), 1.16 (6H, d, ^3^ *J*_H‐H_ = 5.4 Hz, [CH(C*H*_3_)_2_]), 1.14 (6H, d, ^3^ *J*_H‐H_ = 5.5 Hz, [CH(C*H*_3_)_2_]), 1.12 (6H, d, ^3^ *J*_H‐H_ = 6.7 Hz, [CH(C*H*_3_)_2_]), 1.04 (6H, d, ^3^ *J*_H‐H_ = 6.8 Hz, [CH(C*H*_3_)_2_]), 0.88 (18H, s, [C(C*H*_3_)_3_]), 0.11 (3H, s, CH_3_), 0.09 (6H, ov. s, 2 × CH_3_), 0.08 (3H, s, CH_3_); ^13^C NMR (101 MHz, CDCl_3_): δ 162.80 (C_4_, C=O), 162.76 (C_4_, C=O), 158.71 (ArC), 158.68 (ArC), 150.7 (C_2_, C=O), 150.5 (C_2_, C=O), 144.5 (ArC), 144.4 (ArC), 141.6 (C_6_), 141.2 (C_6_), 135.42 (ArC), 135.37 (ArC), 135.3 (ArC), 135.2 (ArC), 130.4 (ArC), 130.33 (ArC), 130.30 (ArC), 130.27 (ArC), 128.34 (ArC), 128.26 (ArC), 128.0 (ArC), 127.9 (ArC), 127.1 (ArC), 117.7 (CH_2_CH_2_ *C*N), 117.4 (CH_2_CH_2_ *C*N), 113.20 (ArC), 113.17 (ArC), 113.15 (ArC), 102.4 (C_5_), 102.1 (C_5_), 87.11 [*C*(Ph)(Ph‐OCH_3_)_2_], 87.07 [*C*(Ph)(Ph‐OCH_3_)_2_], 78.5–78.2 (m, C_2’_), 75.6–75.0 (m, C_3’_), 65.2 (C_1’_), 64.7 (C_1’_), 63.7 (C_5’_), 63.3 (C_5’_), 58.2 (d, ^2^ *J*_C‐P_ = 18.6 Hz, *C*H_2_CH_2_CN), 57.8 (d, ^2^ *J*_C‐P_ = 19.8 Hz, *C*H_2_CH_2_CN), 55.28 (OCH_3_), 55.25 (OCH_3_), 49.5 (C_4’_), 43.3 (d, ^2^ *J*_C‐P_ = 12.7 Hz, [*C*H(CH_3_)_2_]), 43.1 (d, ^2^ *J*_C‐P_ = 12.6 Hz, [*C*H(CH_3_)_2_]), 25.7 [C(*C*H_3_)_3_], 25.6 [C(*C*H_3_)_3_], 24.7–24.5 (m, [CH(*C*H_3_)_2_]), 20.5 (d, ^3^ *J*_C‐P_ = 6.3 Hz, CH_2_ *C*H_2_CN), 20.3 (d, *J* = 6.7 Hz, CH_2_ *C*H_2_CN), 17.93 [*C*(CH_3_)_3_], 17.91 [*C*(CH_3_)_3_], −4.71 (CH_3_), −4.73 (CH_3_), −4.75 (CH_3_), −4.79 (CH_3_); ^31^P{^1^H} NMR (162 MHz, CDCl_3_): δ 149.8 (s), 149.7 (s).

## REAGENTS AND SOLUTIONS

Use distilled, deionized water in all aqueous recipes.

### Brine

To a beaker equipped with a magnetic stirrer bar, add H_2_O (fill approx. two thirds of the beaker). Under continuous stirring, add NaCl (37 g per 100 ml H_2_O) and stir until the majority of the solid has dissolved. Allow any remaining solid to settle to the bottom of the beaker. Transfer the saturated solution to a clean bottle for storage.

### Degassing reaction vessel

Displace the atmosphere of the reaction vessel with nitrogen by placing a nitrogen‐filled balloon attached to a needle into the rubber septum, along with a separate venting needle attached to a gas bubbler. Allow the balloon to empty. Refill the balloon and place it back into the septum and remove the venting needle.

### H_2_SO_4_ (5%, v/v) in ethanol

To a beaker equipped with a magnetic stirrer bar; add ethanol (190 ml) and cool the beaker to 0°C using an ice bath (ice/H_2_O) for 10 min. Under continuous stirring, slowly add concentrated H_2_SO_4_ (10 ml) using a graduated pipette. Allow the mixture to stir at 0°C for a further 5 min. Place the mixture in a wide‐necked container with a screw‐on lid. When sealed correctly, the mixture can be stored at room temperature until it is fully consumed. For disposal, the mixture should be neutralized (using saturated NaHCO_3_) prior to disposal.

### Reaction monitoring

To monitor the reaction with TLC analysis, carefully remove a small aliquot of the reaction mixture (∼10 to 100 μl) and place the aliquot in an Eppendorf. Quench the aliquot as appropriate or dilute it with the same solvent as the reaction (∼500 μl). Spot the quenched/diluted reaction mixture against the starting material; include a co‐spot of the reaction mixture and the starting material. Elute the TLC plate in the stated eluent. Visualize the TLC plate first by UV light (λ = 254 nm) and then by using 5% H_2_SO_4_ in ethanol, followed by heating.

### Recrystallization of 4,4′‐Dimethoxytrityl chloride

Place 4,4′‐dimethoxytrityl chloride (∼3 g) in a 250‐ml round bottom flask with a magnetic stirrer bar and connect the flask to an air condenser. Add ∼80 ml of hexane and heat the suspension to reflux. Once at reflux, add hexane in small portions (∼5 ml) and allow the mixture to reach reflux. Repeat this procedure until all the solid has dissolved. With continued stirring, turn off the heat and allow to reach room temperature. A light‐pink precipitate will start to form. Place the round‐bottom flask in an ice/H_2_O bath and continue to stir for a further 1 h. Assemble a Büchner filtration system; place a filter paper in the Büchner funnel and attach a vacuum pump to the Büchner flask. Turn on the vacuum pump and carefully pour the contents of the round‐bottom flask over the filter paper. With ice‐cold hexane (4 × ∼10 ml), rinse the remaining solid from the round‐bottom flask onto the filter paper. Allow the vacuum to pull the solvent from the solid into the Büchner flask for a further 10 min. Switch the vacuum pump off and transfer the light‐pink solid to a clean 25‐ml round‐bottom flask. Discard the mother liquor. Attach an adapter to the high‐vacuum manifold and place the 25‐ml round‐bottom flask containing the recrystallized 4,4′‐dimethoxytrityl chloride under vacuum for 2 hr. Refill the flask with nitrogen and quickly stopper the flask with a new rubber septum. Wrap the round‐bottom flask in aluminum foil and store at room temperature.

### Saturated NaHCO_3_

To a beaker equipped with a magnetic stirrer bar, add H_2_O (fill approx. two thirds of the beaker). Under continuous stirring, add NaHCO_3_ (11 g per 100 ml H_2_O) and stir until the majority of the solid has dissolved. Allow any remaining solid to settle to the bottom of the beaker. Transfer the saturated solution to a clean bottle for storage.

## Commentary

### Background Information

**Figure 3:**
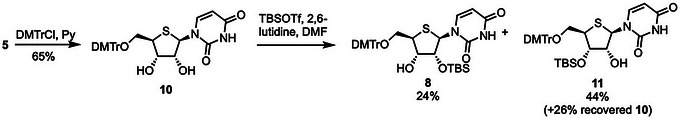
Alternative synthetic approach to regioprotected building block **8**.

At the outset of this investigation, we pursued the synthesis of a 5′‐*O*‐dimethoxytrityl‐protected derivative of **5** (compound **10**, Fig. 3), with the objective of completing regioselective 2′‐*O*‐silylation, as the entry to **8**, reported by Egli and others (Haeberli et al., 2005; Leydier et al., 1995) Unfortunately, we were unable to replicate this reported regioselctivity in accessing the required 2′‐*O*‐protected material **8** for subsequent phosphitylation. We obtained consistently low yields (24% was the best) alongside larger amounts of the undesired 3′‐*O*‐TBS regioisomer **11** (44%). We evaluated several combinations of silylation conditions (including those reported, TBDMSCl/AgNO_3_) and scales (mg to g), but with no overall improvement. Although these regioselectivities are not significantly different from those reported by Egli (38% for **8** and 21% for **11**), we decided instead to explore the route presented herein using the 3,5‐di‐*tert*‐butylsilylene‐system introduced by Beigelman (Serebryany & Beigelman, 2002). Even though this includes additional synthetic steps to access **9** from **5** (four steps vs. two for regioselective 3′‐*O*‐silylation approach), there are no issues regarding complex chromatographic regioisomer separation and thus no possibilities of incorrect linkage formation during any subsequent oligonucleotide synthesis.

In conclusion, and to the best of our knowledge, this study demonstrates the first example utilizing this protecting group strategy to access a 4′‐thiouridine amidite building block for automated assembly. Each of the synthetic steps (to access **9** from **5**) offers good yields and we envisage that this approach can be extrapolated to the synthesis of other nucleosides (C, G, A) containing a 4′‐thioribose unit.

### Critical Parameters and Troubleshooting

In terms of difficulty, the experimental procedures described in the above protocol range from simple deprotection steps to more complex techniques requiring extra precautions such as anhydrous reaction setups, the use of degassed solvents, and the use of hazardous substances. Where appropriate, further guidelines and necessary precautions have been included within the protocol.

In our experience, reactions with 4′‐thionucleosides generally take longer than reactions their 4′‐oxo counterparts, evident when performing the 5′‐*O*‐tritylation. We found it necessary to use only freshly recrystallized DMTrCl to achieve acceptable yields.

### Time Consideration

Most of the reactions described herein are short and take only a few hours once the reaction is started, with the slowest reaction being the tritylation (from **7** to **8**), requiring 18 hr to achieve agreeable yields. With efficient planning, the complete synthesis from **1** to **9** can be completed in 10 to 12 days; however, it would be pertinent to set aside at least 15 days to account for any unexpected results or requirement of further purification.

### Anticipated Results

Some experience is required to achieve acceptable yields for the nucleobase installation and phosphitylation steps, and initial efforts are likely giving more modest yields (e.g., <50% for the two steps to synthesize **3** and <50% for the phosphitylation). With experience, the yields reported can be expected.

### Author Contributions

**Caecilie Benckendorff**: Formal analysis; investigation; methodology; writing – original draft; writing – review & editing. **Yogesh S. Sanghvi**: conceptualization; writing – review & editing. **Gavin J. Miller**: conceptualization; funding acquisition; project administration; supervision; writing – original draft; writing – review & editing.

### Conflict of Interest

The authors declare no conflict of interest.

## Supporting information


*The data that support this protocol (1H/13C/31P NMR and HRMS) are available in the supplementary material of this article*.

## Data Availability

The data that supports the findings of this study are available in the supplementary material of this article.
