# Supplementary material for: Preparation of a 4′‐Thiouridine Building‐Block for Solid‐Phase Oligonucleotide Synthesis
Source: Curr Protoc. 2023 Sep 25;3(9):e878. doi: 10.1002/cpz1.878 (PMC10946921; doi:10.1002/cpz1.878)

Preparation of a 4’-thiouridine building-block for solid-phase oligonucleotide synthesis

Caecilie M. M. Benckendorff,[a,b] Yogesh S. Sanghvi[c] and Gavin J. Miller[a,b*]

[a]Centre for Glycoscience, Keele University, Keele, Staffordshire, ST5 5BG, UK

[b]Lennard-Jones Laboratory, School of Chemical and Physical Sciences, Keele University, Keele, Staffordshire, ST5 5BG, UK

[c]Rasayan Inc., 2802, Crystal Ridge Road, Encinitas, CA92024-6615, California, USA

Corresponding author: g.j.miller@keele.ac.uk

Supporting Information

Representative NMR spectra for compounds **2-9**

(2R,3S,4R)-2,3-*O*-Isopopropylidene-5-*O*-*tert*-butyldiphenylsilyl-1-(4-sulfinyl)cyclopentane

^{1}H NMR (400 MHz, $CDCl_3$)

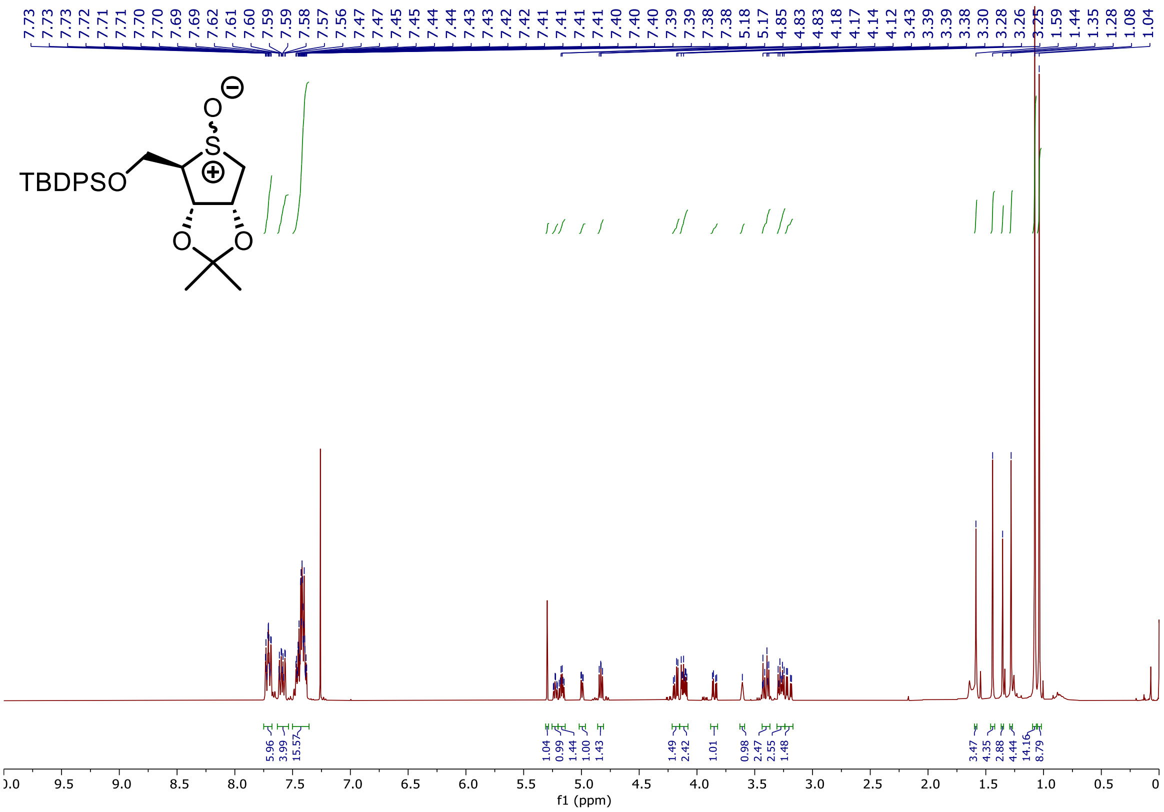

^{13}C NMR (101 MHz, $CDCl_3$)

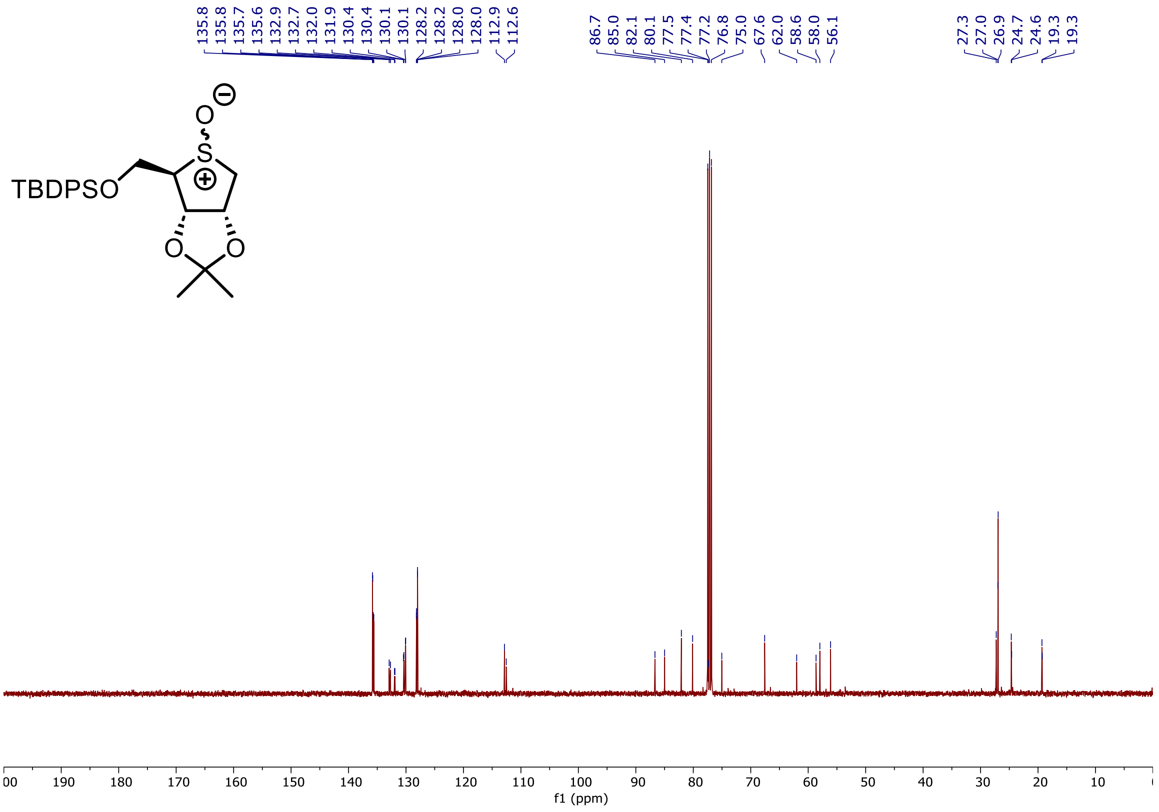

2',3'-*O*-Isopropylidene-5'-*O*-*tert*-butyldiphenylsilyl-4'-thiouridine

^{1}H NMR (CDCl$_3$)

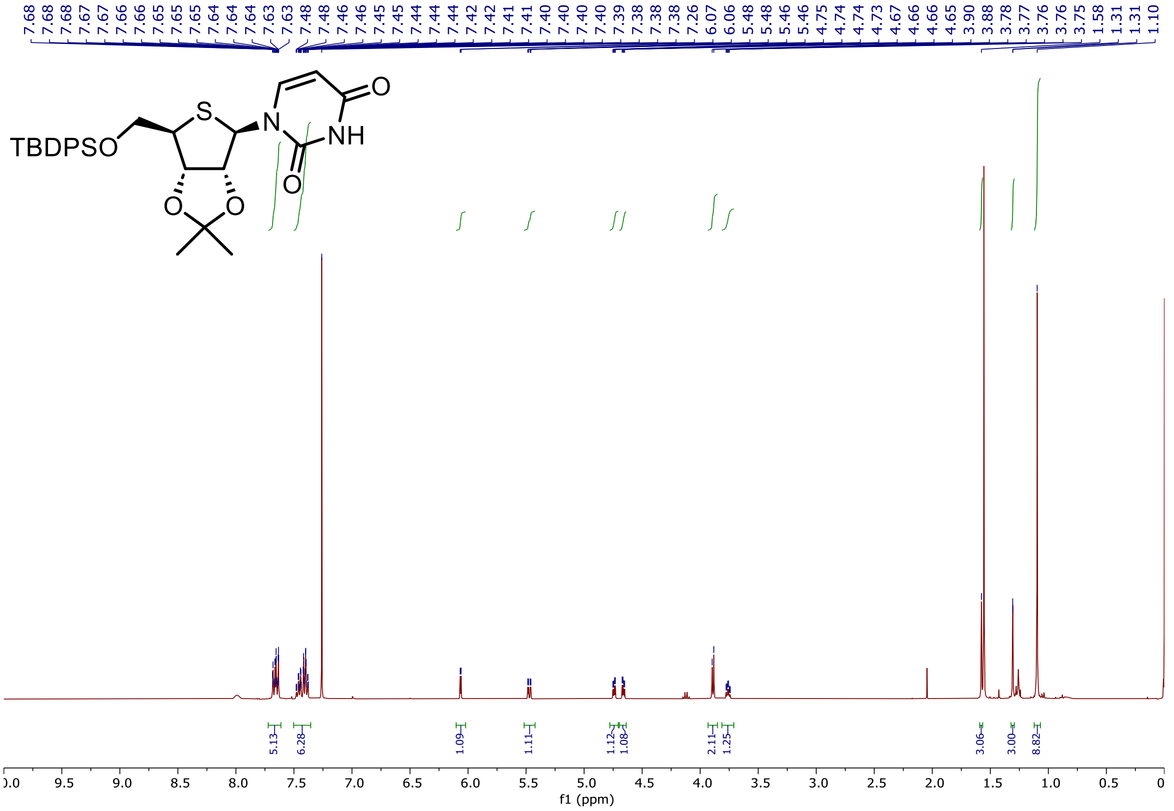

^{13}C MR (400 MHz, CDCl$_3$)

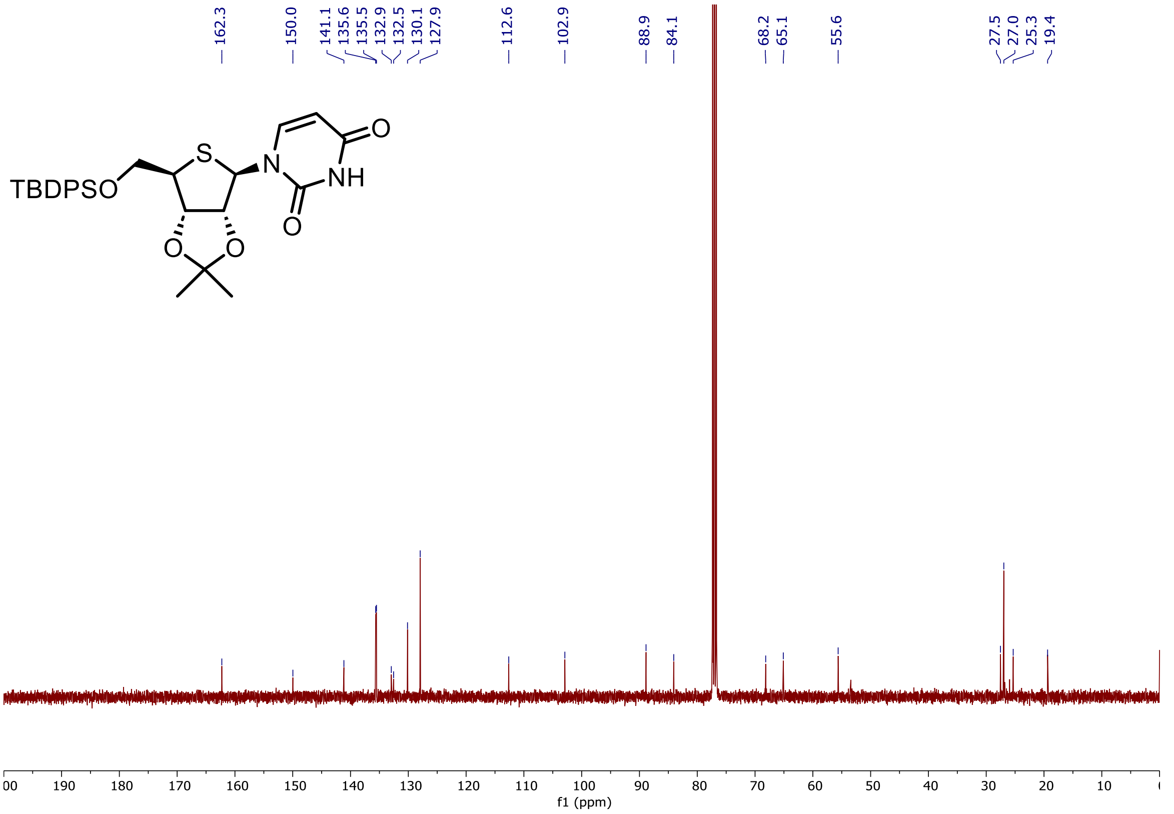

2’-3’-*O*-Isopropylidene-4’-thiouridine

^{1}H NMR (400 MHz, $CDCl_3$)

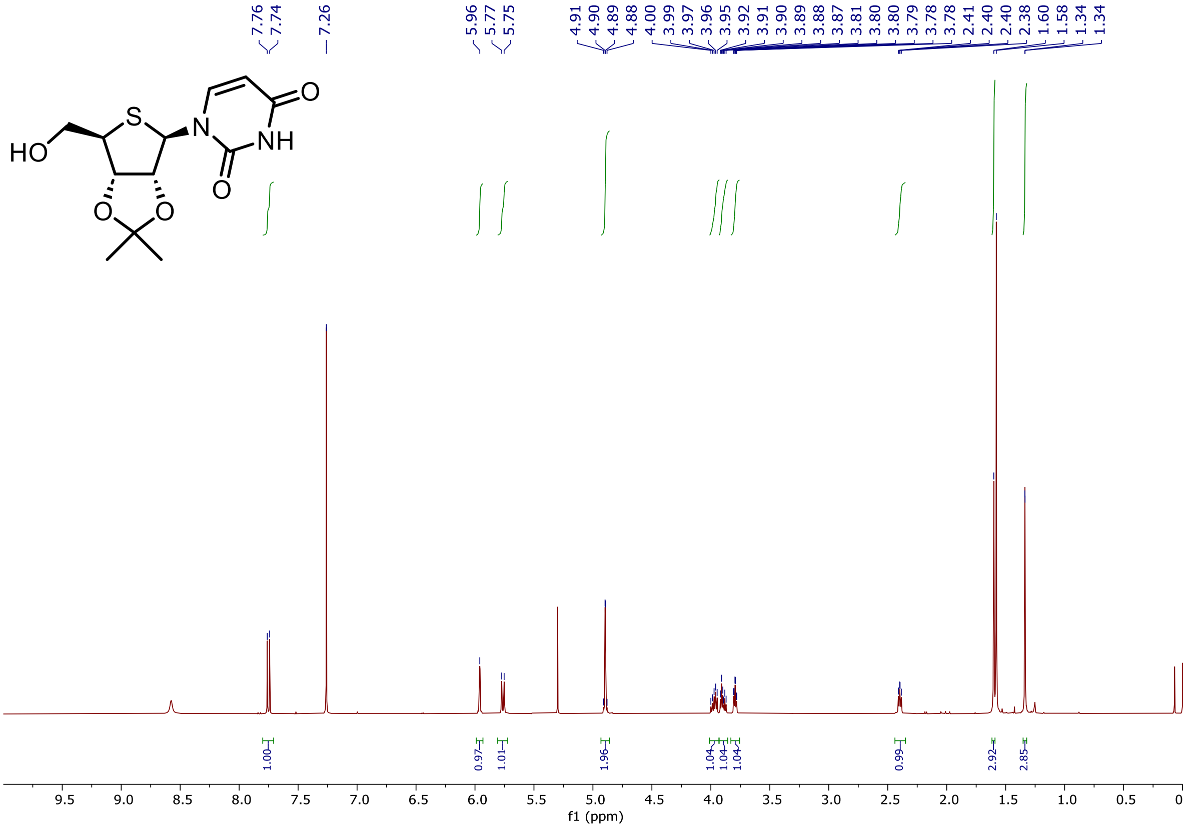

^{13}C NMR (101 MHz, $CDCl_3$)

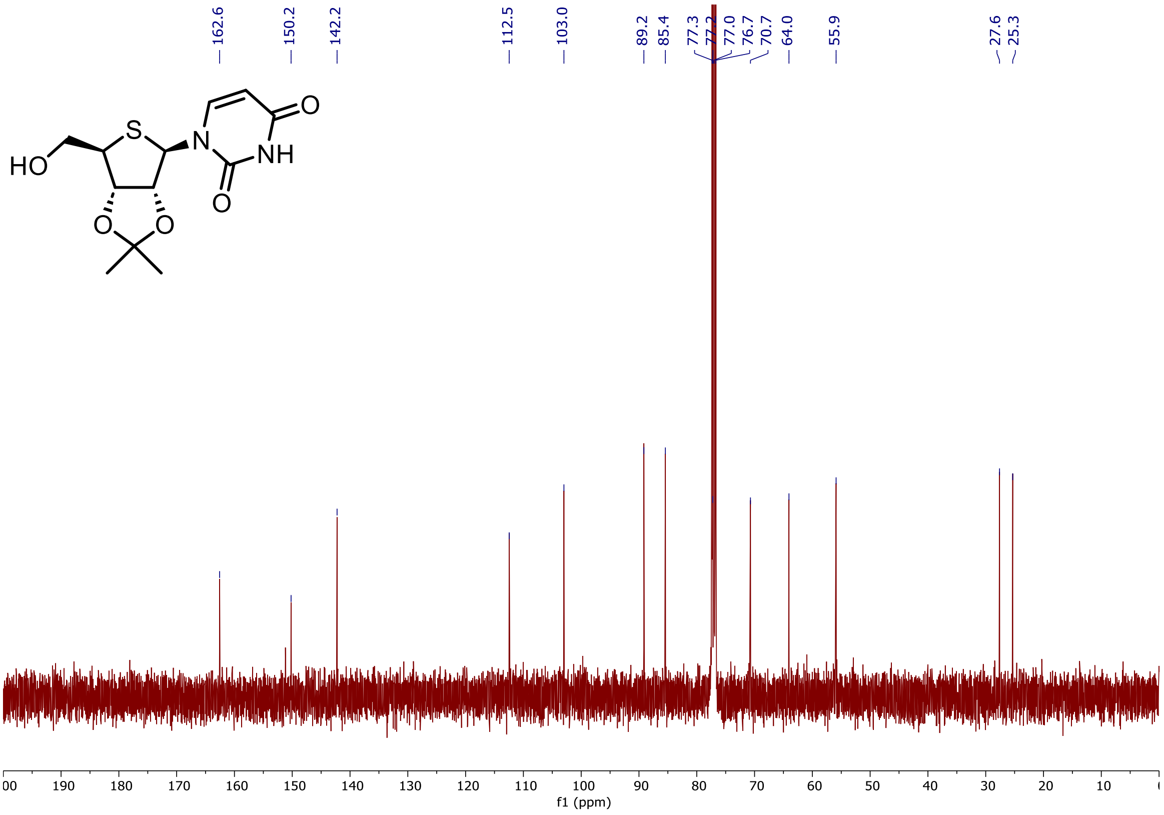

4’-Thiouridine

^{1}H NMR (400 MHz, D_2O):

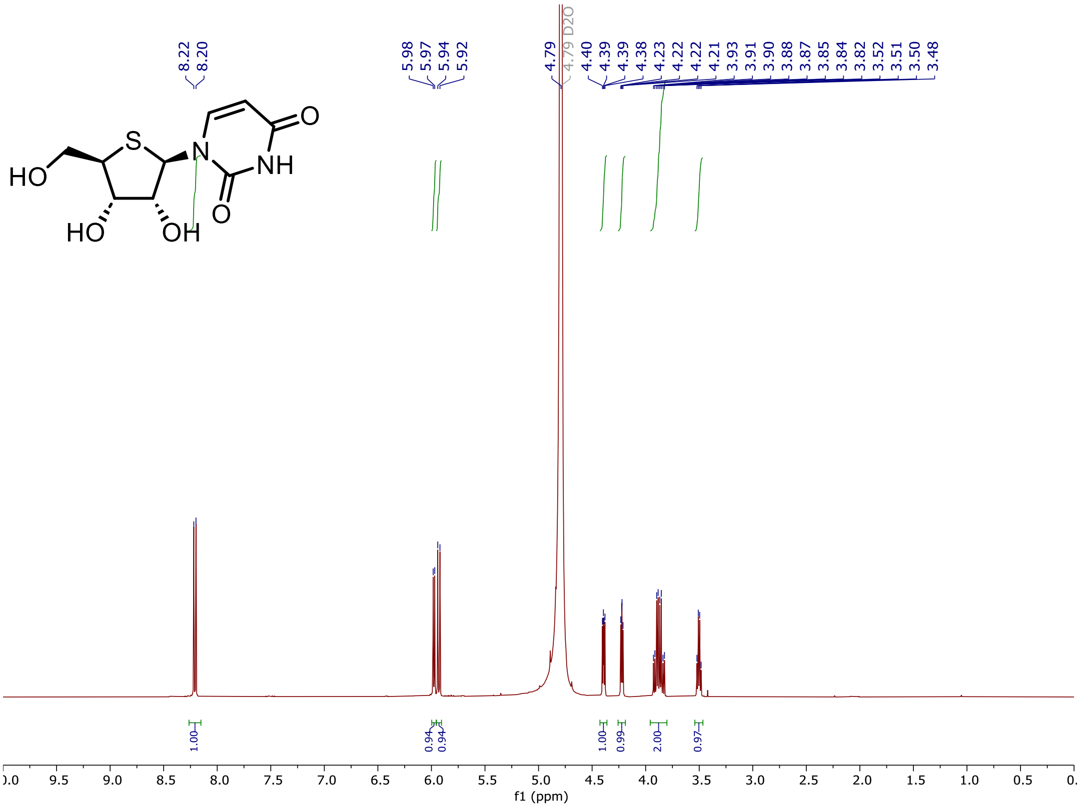

^{13}C NMR (101 MHz, D_2O):

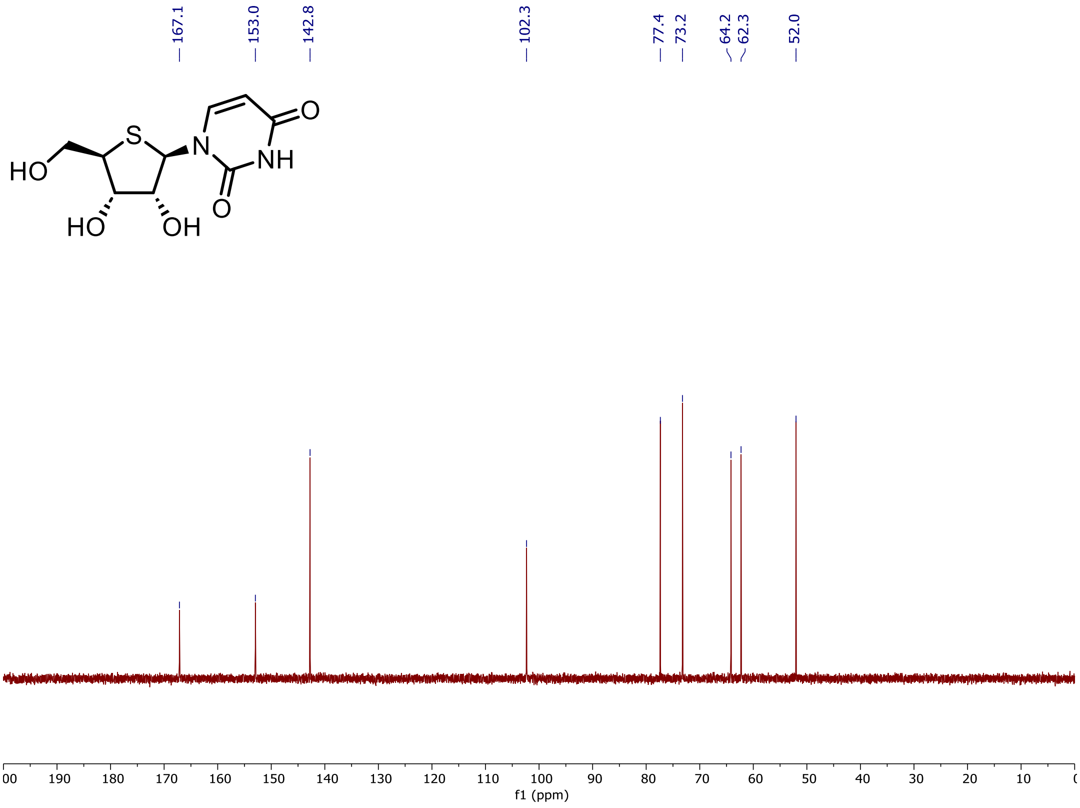

2’-*O*-*tert*-Butyldimethylsilyl-3’,5’-di-*tert*-butylsiloxy-4’-thiouridine

^{1}H NMR (400 MHz, $CDCl_3$)

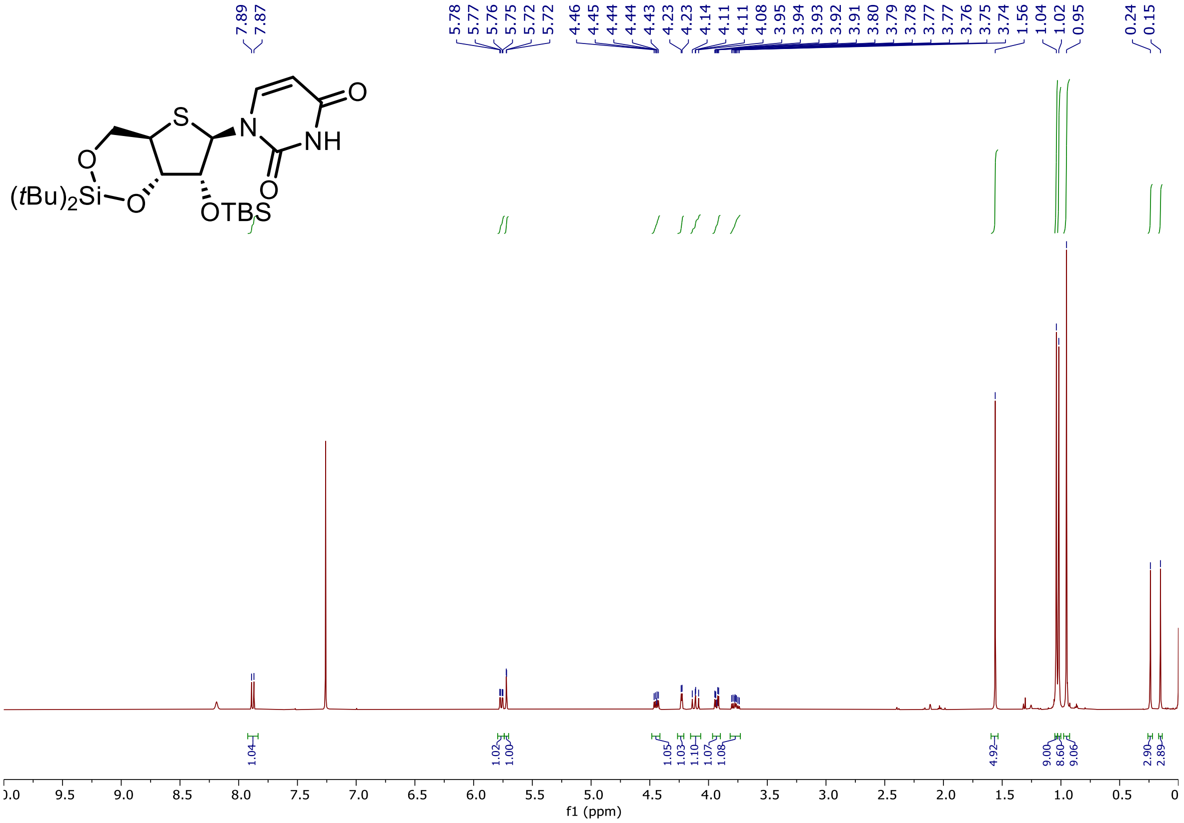

^{13}C NMR (101 MHz, $CDCl_3$)

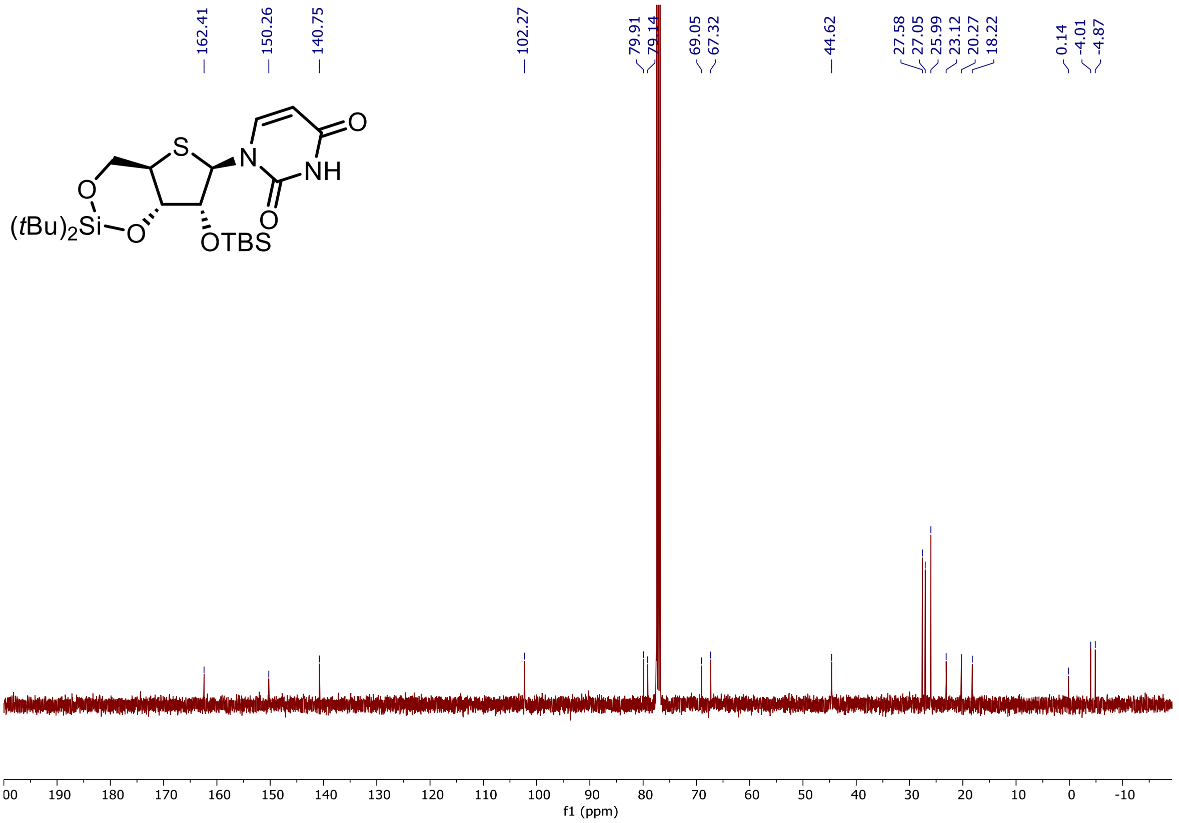

2’-*O*-*tert*-Butyldimethylsilyl-4’-thiouridine

^{1}H NMR (400 MHz, MeOD)

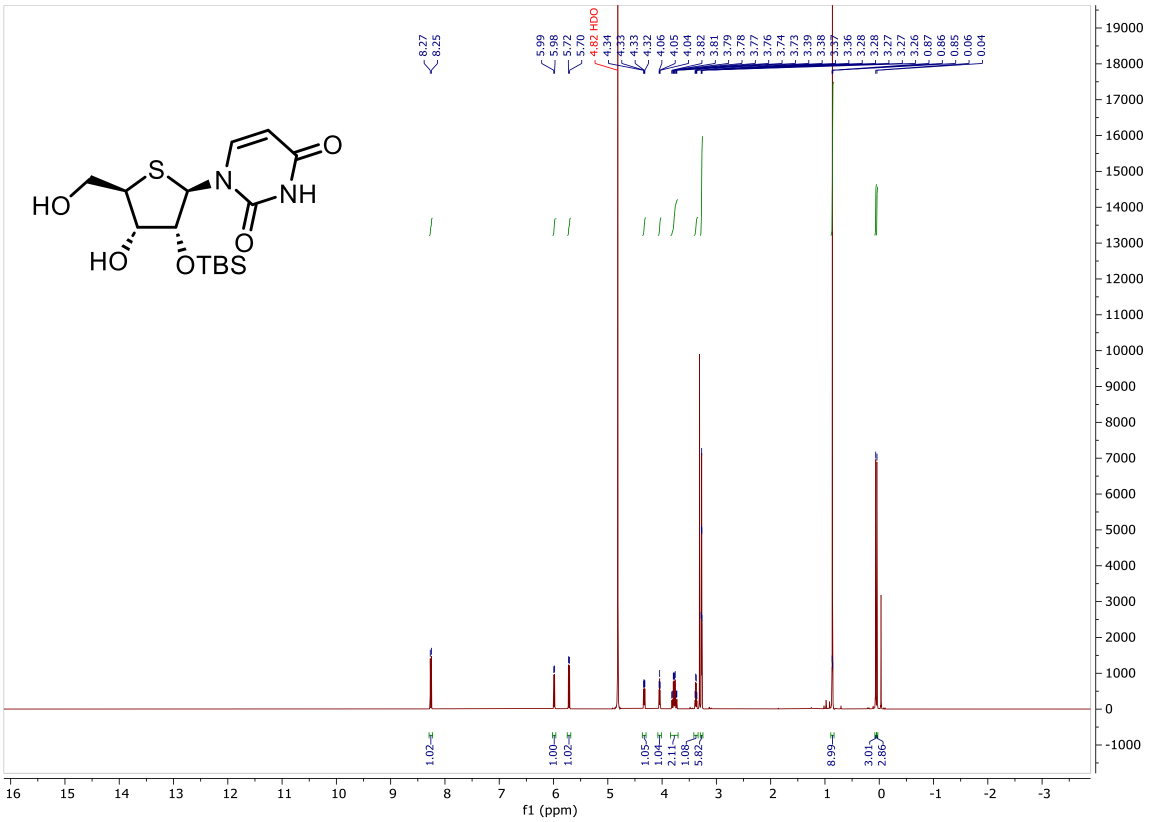

^{13}C NMR (101 MHz, MeOD)

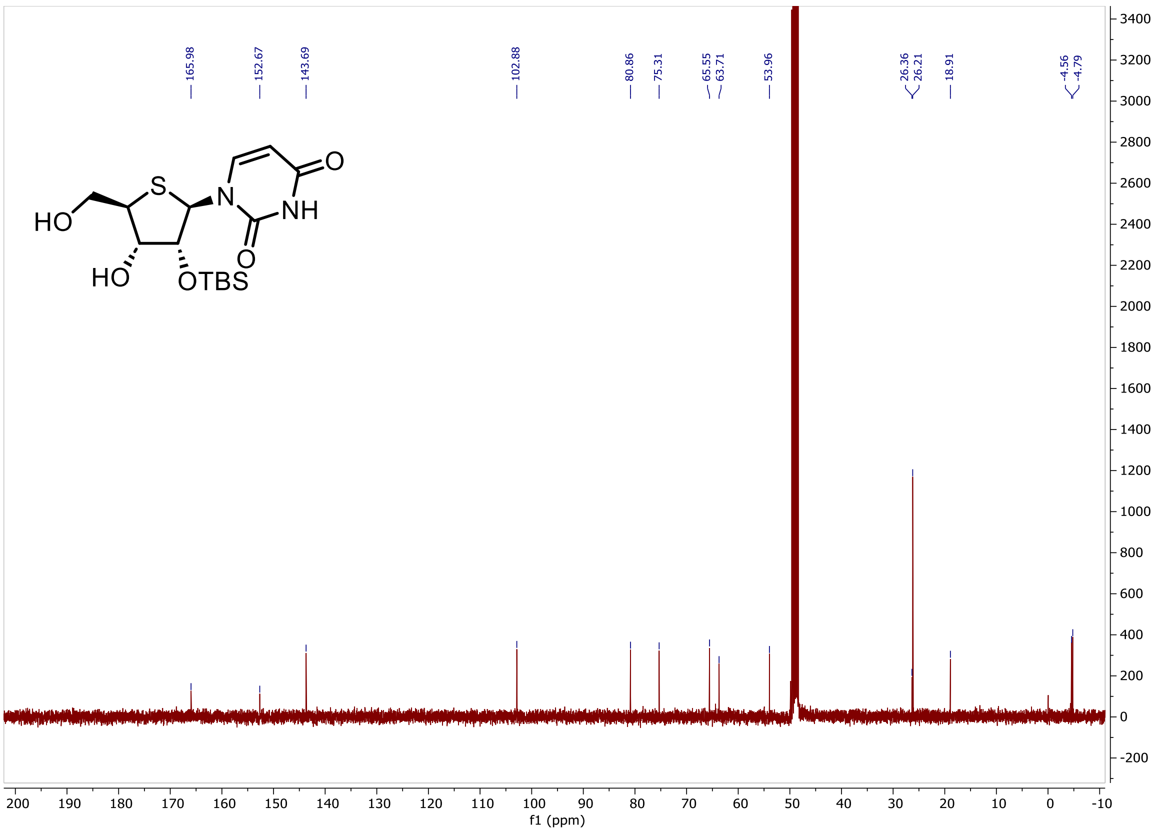

2’-*O*-*tert*-Butyldimethylsilyl-5’-*O*-dimethoxytrityl-4’-thiouridine

^{1}H NMR (400 MHz, $CDCl_3$)

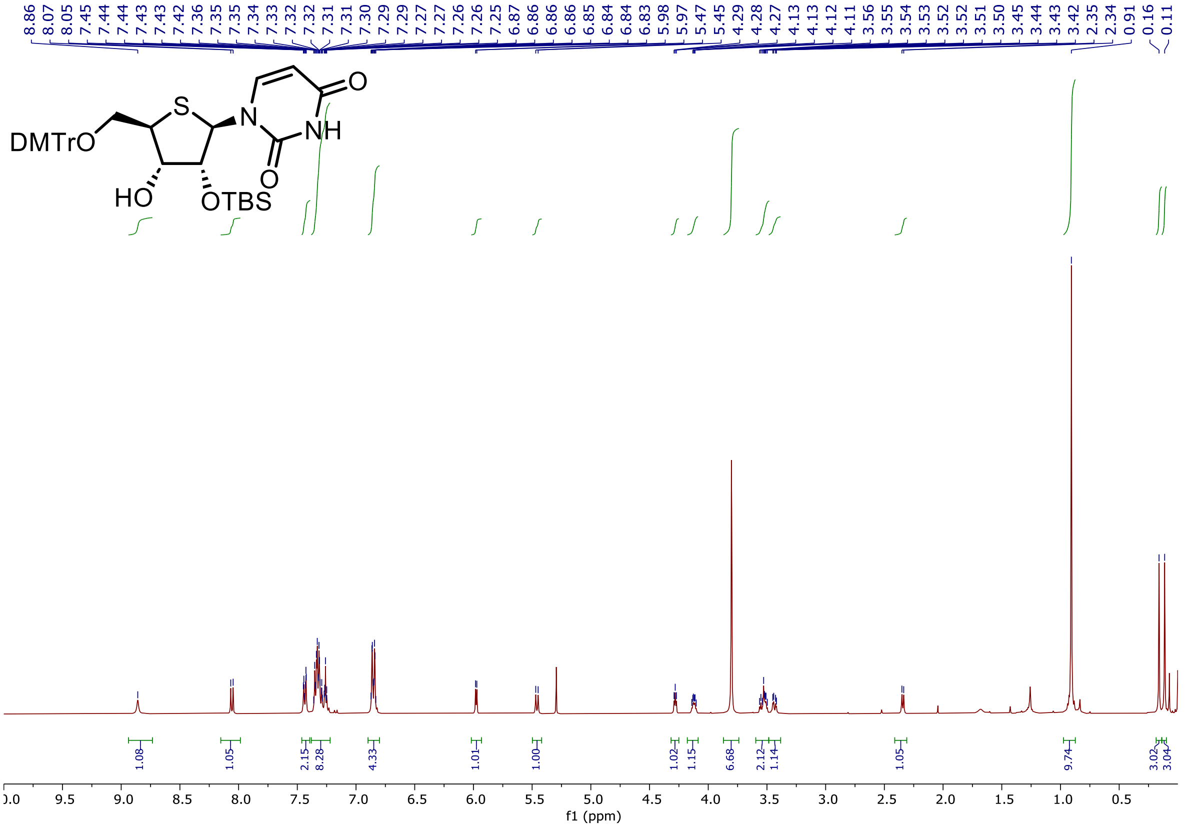

^{13}C NMR (400 MHz, $CDCl_3$)

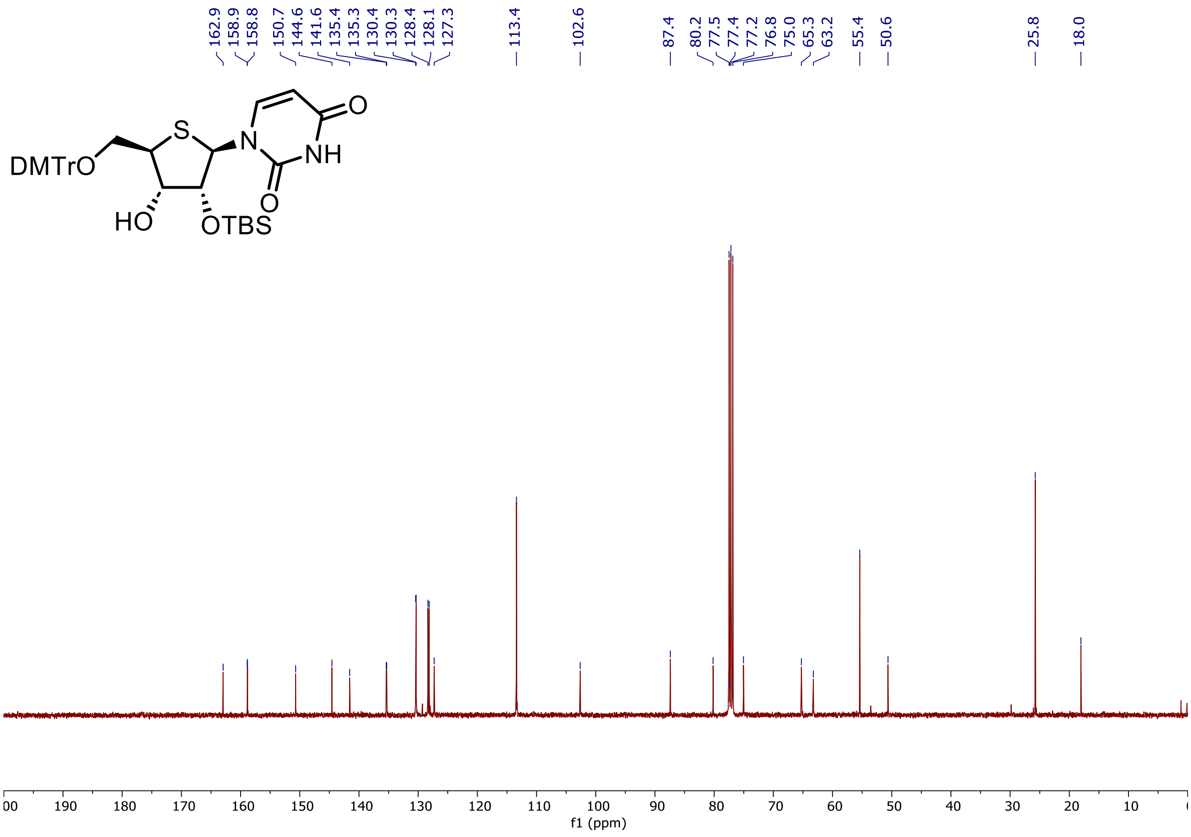

2'-*O*-*tert*-butyldimethylsilyl-3'-*O*-[(2-cyanoethoxy)(*N*,*N*-diisopropylamino)phosphino]-5'-*O*-dimethoxytrityl-4'-thiouridine

^{1}H NMR (400 MHz, $CDCl_3$):

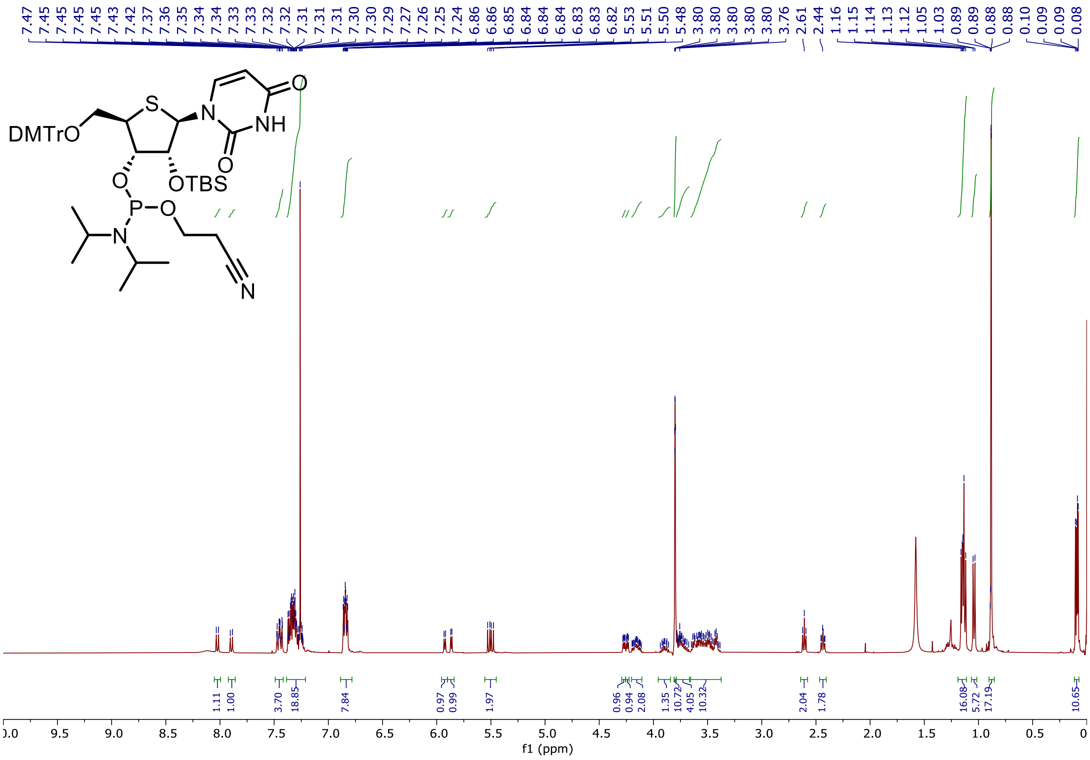

^{13}C NMR (101 MHz, $CDCl_3$):

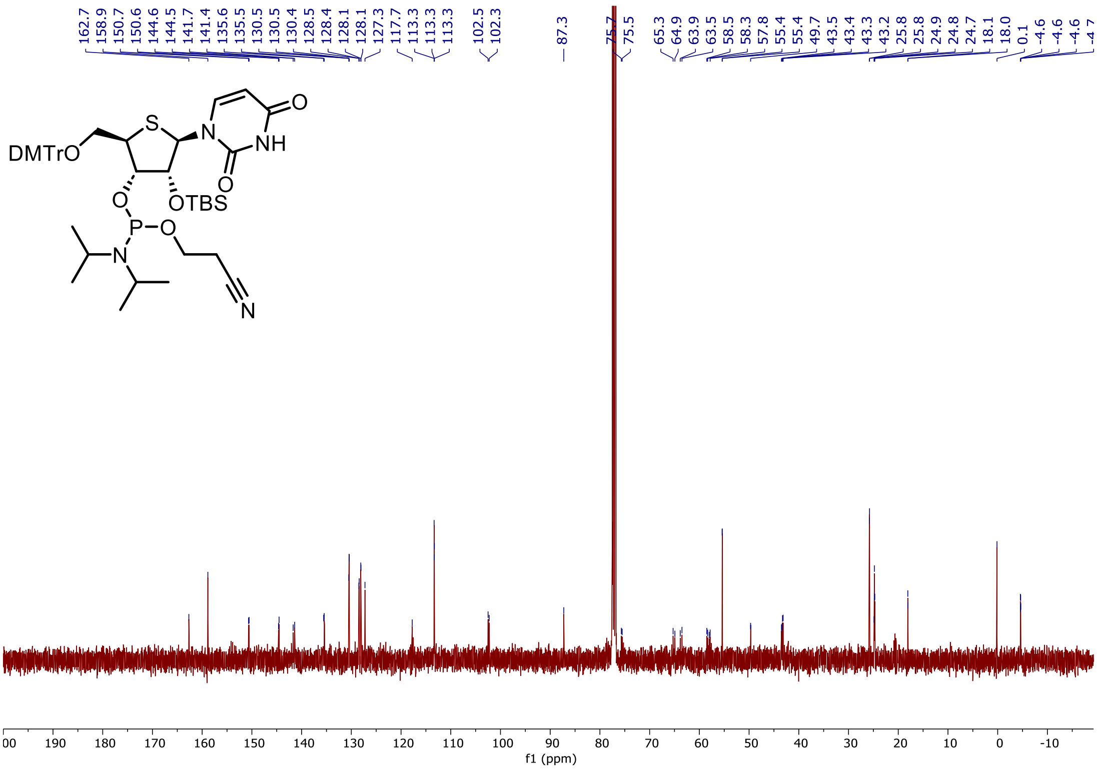

^{31}P NMR (162 MHz, $CDCl_3$):

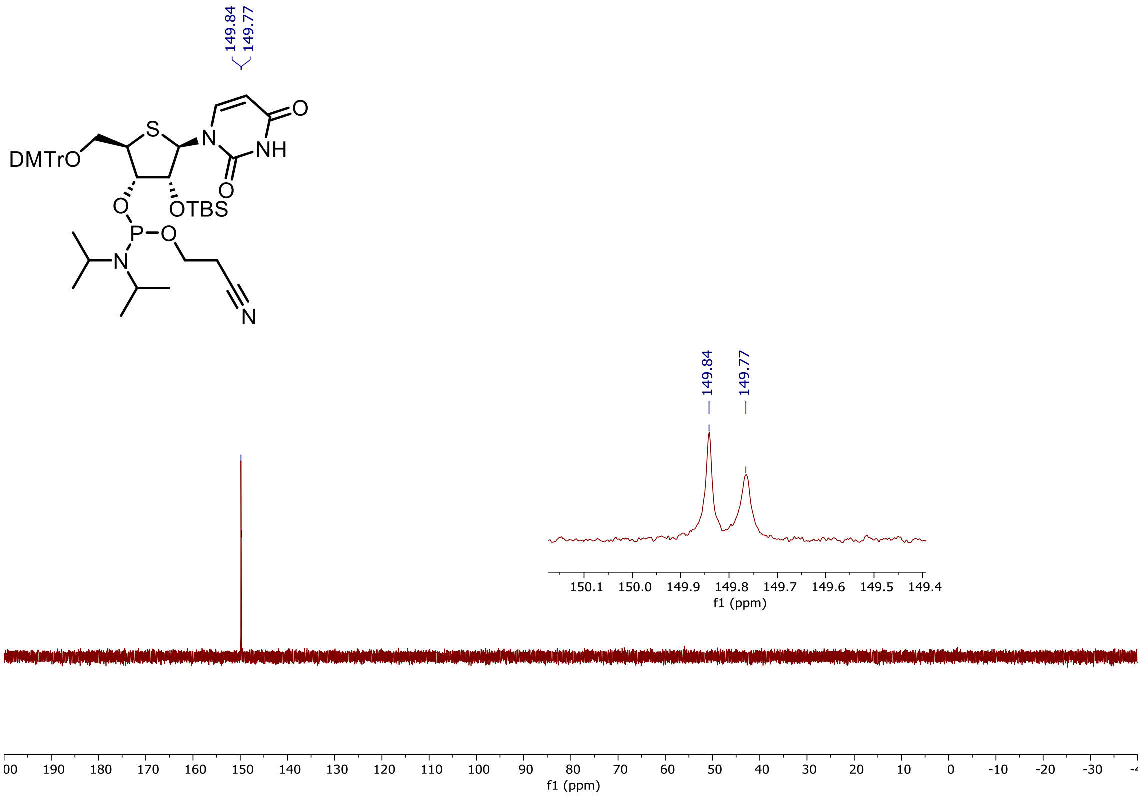

Supplement: Supplementary file 1 — The data that support this protocol (1H/13C/31P NMR and HRMS) are available in the supplementary material of this article. [file CPZ1-3-0-s001.pdf]
